# Complete Blood Count-Derived Inflammatory Markers in Pediatric Lower Respiratory Tract Infection: A Narrative Review

**DOI:** 10.3390/pathogens15090956

**Published:** 2026-09-08

**Authors:** Joan Joan, Clarissa Chandra, Clara Annabel Wibisono, Hans Davis Gunawan, Vivian Chang, Anton Sumarpo

**Affiliations:** 1 Medical Profession Program, Faculty of Medicine, Maranatha Christian University, Bandung 40164, Indonesia; joann.150505@gmail.com; 2 Undergraduate Medical Program, Faculty of Medicine, Maranatha Christian University, Bandung 40164, Indonesia; icaa.chandra@gmail.com (C.C.); claraannabelw@gmail.com (C.A.W.); 28vivianchang@gmail.com (V.C.); 3 Undergraduate Biotechnology Program, Faculty of Medicine, Maranatha Christian University, Bandung 40164, Indonesia; ghansdavis@gmail.com; 4 Department of Clinical Pathology, Faculty of Medicine, Maranatha Christian University, Bandung 40164, Indonesia

**Keywords:** complete blood count, neutrophil-to-lymphocyte ratio, lower respiratory tract infection, pneumonia, child, immunopathogenesis

## Abstract

Lower respiratory tract infections (LRTIs) are a leading infectious cause of death in children worldwide. Complete blood count (CBC)-derived inflammatory ratios are low-cost and widely available, yet their reported performance varies. We reviewed 46 PubMed-indexed studies (2016–2026) in children aged 0–18 years with viral, bacterial, or mycobacterial LRTI. The findings were mapped across diagnosis, etiological differentiation, severity, and outcome prediction. The neutrophil-to-lymphocyte ratio (NLR) was the most investigated marker, with the broadest evidence base for severity assessment and for separating bacterial from viral disease, although its performance remained pathogen-, population-, and endpoint-dependent; it was elevated in severe pertussis, tuberculosis, *Mycoplasma pneumoniae*, *Streptococcus pneumoniae*, and SARS-CoV-2 infection, whereas findings in respiratory syncytial virus infection were conflicting. The platelet-to-lymphocyte ratio had the next broadest evidence base for severity, and the systemic immune-inflammation index was consistently elevated in *Mycoplasma pneumoniae* and SARS-CoV-2 infection. No single ratio performed consistently across all etiologies. Ratios governed by one dominant immunopathogenic mechanism behaved predictably; those whose numerator and denominator respond to the same process did not. Cut-offs were neither standardized, age-specific, nor externally validated, and most estimates derived from retrospective cohorts in few countries; reported values therefore represent association and discrimination rather than validated prediction. Standardized, age-specific thresholds are needed before these markers can support routine risk stratification.

## 1. Introduction

Lower respiratory tract infections (LRTIs) refer to infections caused by pathogens affecting the respiratory tract below the larynx [1]. In 2023, LRTIs were responsible for an estimated 2.50 million deaths worldwide, with the burden concentrated at the extremes of age, in children younger than 5 years and in older adults [2]. Low-cost and widely available markers with adequate diagnostic and prognostic performance are therefore needed to support early recognition and risk stratification.

Blood-based inflammatory markers obtained from complete blood count have recently been investigated in the clinical management of LRTI cases in children aged 0 to 18 years. Complete blood count (CBC) provides data on different leukocyte types, such as basophils, eosinophils, neutrophils, monocytes, and lymphocytes. Ratios derived from different leukocyte populations can reflect the inflammatory response across a wide range of diseases [3]. CBC-derived inflammatory markers have been applied in numerous studies for diagnosis, etiological differentiation, severity assessment, and outcome prediction. However, an updated synthesis of the value of CBC-derived inflammatory markers is still lacking in pediatric populations with LRTIs. Therefore, a narrative review is warranted to summarize current evidence and identify potential clinical applications of CBC-derived inflammatory markers.

This review has three objectives: (i) to summarize the reported performance of CBC-derived inflammatory markers in children aged 0 to 18 years with LRTI according to four distinct clinical questions, namely diagnosis of infection, etiological differentiation, severity assessment, and outcome prediction; (ii) to examine whether the direction and consistency of each marker can be explained by the immunopathogenesis of the underlying pathogen; and (iii) to identify the methodological gaps, including the absence of standardized, age-specific, and externally validated cut-offs, that currently prevent routine clinical implementation. The reviewed studies include pediatric LRTI with diverse etiologies, comprising bacterial, mycobacterial, viral, and mixed-pathogen infections.

## 2. Clinical Performance of CBC-Derived Inflammatory Markers

### 2.1. Evaluated Inflammatory Ratios Derived from CBC

CBC-derived inflammatory ratios obtained in pediatric LRTI are listed in Table 1 below.

### 2.2. Study Characteristics of Pediatric LRTI

This narrative review is based on a structured, non-systematic literature search of PubMed. The search was not conducted as a systematic or scoping review: no protocol was registered, no formal risk-of-bias instrument was applied, and screening and data extraction were not performed in duplicate. Applying the pragmatic criteria below, the search returned 48 candidate articles, of which two were set aside after full-text reading (incomplete data, *n* = 1; ineligible study population, *n* = 1), leaving 46 primary studies. The keywords used in this study include pediatrics, LRTI, relevant etiologies, and blood-based inflammatory markers. The search strategies for the PubMed database are provided in full as Table S8 of the Supplementary Materials. Data were extracted and compiled for the review from April to August 2026. Eligible articles were original studies published between September 2016 and May 2026, written in English, and available in full text. May 2026 was set as the closing date of the publication window so that indexing would be complete for every eligible month; articles published after that date were therefore not considered, irrespective of indexing status. The final PubMed search was conducted on 9 August 2026. Meanwhile, studies that did not evaluate CBC-derived inflammatory markers, were unrelated specifically to LRTI, used a case report design, or involved animal or in vitro studies were excluded. For the purposes of this review, pediatric LRTI was operationally defined as clinically or microbiologically diagnosed infection of the airways below the larynx—including tracheitis, bronchitis, bronchiolitis, bronchopneumonia, and pneumonia as etiologically defined LRTI syndromes—in children aged 0 to 18 years. Studies enrolling broader respiratory infection populations were eligible only when results for an LRTI population or a defined LRTI subgroup were reported separately, and studies addressing complications of LRTI (for example, parapneumonic effusion, empyema, plastic bronchitis, or sepsis complicating pneumonia) were eligible when the underlying condition was an LRTI. In most included studies, CBC parameters were obtained at initial presentation or on hospital admission; the interval between symptom onset and blood sampling, and the relation of sampling to treatment initiation, were rarely reported.

The included studies covered bacterial, mycobacterial, and viral etiologies of pediatric LRTI, grouped here by microbiological characteristics as bacterial, viral, and mixed infections. Mixed bacterial infection denotes study populations comprising more than one bacterial pathogen or bacterial pneumonia without a single specified organism; mixed viral infection denotes populations comprising more than one viral pathogen; and mixed pathogen infection denotes populations in which bacterial and viral etiologies were combined or compared within a single cohort. These categories describe the composition of the study populations rather than microbiologically confirmed co-infection, since the detection methods used in most primary studies could not distinguish true infection from colonization or incidental carriage. The characteristics of the 46 included studies are summarized in Table 2. These 46 articles are the primary studies from which all extracted data are drawn; the remaining references cited in this review are background, mechanistic, or epidemiological sources and are not part of the 46 included studies. These 46 articles are the primary studies from which all extracted data are drawn; the remaining references cited in this review are background, mechanistic, or epidemiological sources and are not part of the 46.

**Figure 1:**
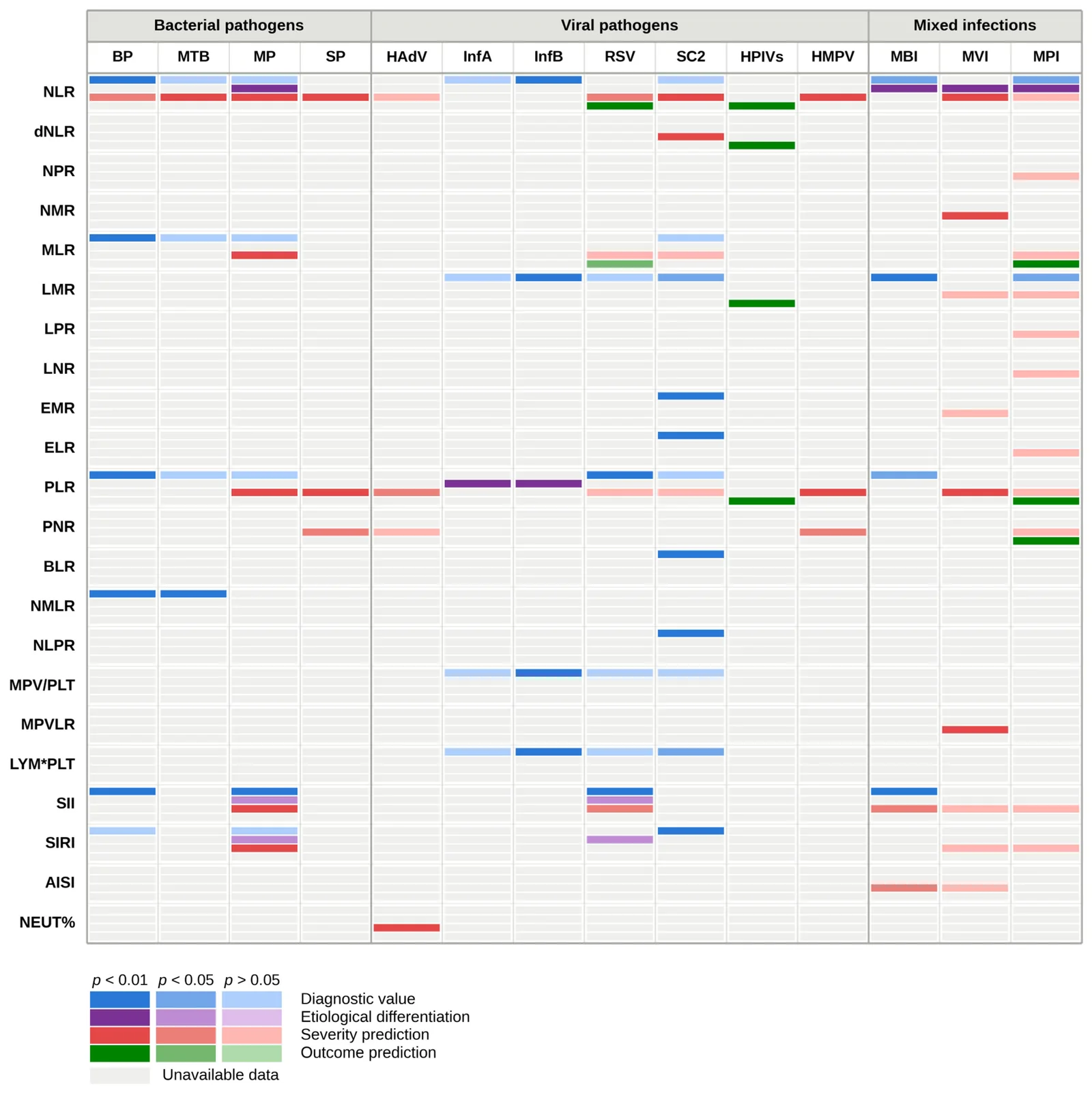
Reviewed CBC-derived inflammatory markers for etiologies of pediatric LRTI. BP = *Bordetella pertussis*; MTB = *Mycobacterium tuberculosis*; MP = *Mycoplasma pneumoniae*; SP = *Streptococcus pneumoniae*; HAdV = human adenovirus; InfA = Influenza A; InfB = Influenza B; RSV = respiratory syncytial virus; SC2 = SARS-CoV-2; HPIVs = human parainfluenza viruses; HMPV = human metapneumovirus; MBI = mixed bacterial infection; MVI = mixed viral infection; MPI = mixed pathogens infection.

This paper synthesized etiologies by grouping bacteria based on cell-wall structure, and viruses by genome type and envelopes. Findings were mapped across diagnosis, etiology, severity, and outcome as depicted in Figure 1.

### 2.3. Performance of CBC-Derived Inflammatory Markers in Bacterial LRTI

NLR, MLR, PLR, NMLR, SII, and SIRI were evaluated in the included studies as diagnostic tools. Based on the studies, PLR was consistently and significantly decreased in children with pertussis. NMLR also consistently increased in children diagnosed with pertussis. Meanwhile, the results for NLR, MLR, and SII varied across studies. SII increased significantly in one study but showed no significant difference between pertussis and non-pertussis groups in another study [5]. NLR demonstrated potential for assessing disease severity in pertussis [6].

In cases of MTB infection, NLR, MLR, NMLR, and PLR were used for diagnosis and to assess disease severity. Meanwhile, NLR showed a significant increase as the severity of tuberculosis increased [7,8,9,10].

MP infections are among the most common in pediatric patients. SII consistently increased in PCR-positive cases of MP. However, NLR, PLR, MLR, SII, and SIRI tended to increase with the severity of MP infection. The same elevated markers, except MLR, have been used to distinguish MP from other infections [6,11,13,14,16,17,19,20,22,24].

SP infection is also frequently observed in pediatric patients. NLR, PLR, and PNR were associated with SP severity. In severe SP infection cases, NLR and PLR increased, whereas PNR decreased [21]. The bacterial studies are listed in Table S5. There, the bacterial pathogens are grouped by cell wall structure: *Bordetella pertussis* as a Gram-negative coccobacillus, *Mycobacterium tuberculosis* as an acid-fast bacillus, *Mycoplasma pneumoniae* as a cell wall-deficient atypical bacterium, and *Streptococcus pneumoniae* as a Gram-positive diplococcus.

Based on the included studies, the NLR was the most investigated inflammatory marker in both bacterial and viral LRTI, with reported optimal cut-off values ranging from 0.43 to 2.69, sensitivities up to 85.40%, and specificities up to 86%. This range is a descriptive summary of heterogeneous studies and not a clinically interpretable interval: the individual cut-offs were derived in different pathogens, age groups, comparator populations, and endpoints, using different laboratory units and sampling times, and the values are therefore not interchangeable. Higher NLR was generally associated with more severe disease in several bacterial and viral infections, including Mycoplasma pneumoniae, Mycobacterium tuberculosis, Streptococcus pneumoniae, RSV, and SARS-CoV-2. However, its diagnostic performance was not consistent across all pathogens, suggesting that NLR is more useful when interpreted alongside the patient’s clinical presentation.

Unlike NLR, MLR did not show a consistent pattern across bacterial LRTIs. Some studies reported higher MLR in active tuberculosis and severe *Mycoplasma pneumoniae* infection, with reported cut-off values ranging from 0.10 to 0.378, reaching 80.20% sensitivity and 87.50% specificity, whereas others found no significant difference between patient groups. This variation may reflect the different roles of monocytes during chronic and acute bacterial infections.

Compared with other composite inflammatory markers, SII showed more consistent findings, particularly in *Mycoplasma pneumoniae* infection. Higher SII values were reported in PCR-confirmed cases and in children with more severe disease, while several studies also found that SII could help differentiate *Mycoplasma pneumoniae* from RSV. However, similar findings were not consistently observed in pertussis, suggesting that the clinical value of SII may vary across bacterial pathogens.

The available evidence for SIRI, NMLR, and PNR is still limited. NMLR was mainly investigated in pertussis and showed encouraging diagnostic performance, whereas PNR was associated with disease severity in pneumococcal pneumonia. Reported optimal cut-off values for PLR ranged widely from 71.26 to 143.621. Despite this variability, PLR achieved sensitivity of up to 88.36% and specificity up to 95%. Further studies involving a wider range of bacterial pathogens are needed before these markers can be recommended for routine clinical use. The detailed findings of each marker are presented in Table 3.

### 2.4. Performance of CBC-Derived Inflammatory Markers in Viral LRTI

CBC-derived inflammatory markers have also been investigated in viral LRTI. NLR and PNR did not reliably discriminate HAdV severity. For HAdV severity evaluation, elevated PLR showed better discrimination [22,23].

NLR, PLR, and PNR have also been used as indicators of HMPV severity. However, NLR was reduced in severe cases of community-acquired pneumonia with HMPV infection [24,25].

NLR, LMR, MPV/PLT, and LYM*PLT contributed to the diagnosis of Influenza B. Elevated NLR and MPV/PLT were associated with confirmed Influenza B infection, whereas reduced LMR and LYM*PLT were also associated with Influenza B. In contrast, the same markers exhibited poor diagnostic performance for Influenza A [39,40].

In cases of HPIVs infection, NLR, dNLR, PLR, and LMR were used to predict the risk of PICU admission and mechanical ventilation. Pediatric patients admitted to the PICU showed significantly higher NLR, dNLR, and PLR values, along with significantly lower LMR values [26].

Current evidence suggests that lower PLR and SII values were associated with RSV infection, whereas other markers generally did not demonstrate significant differences between RSV-infected patients and healthy groups. Beyond diagnosis, several biomarkers have been explored in relation to disease severity. A significantly elevated NLR has been associated with an increased risk of pediatric intensive care unit (PICU) admission. Furthermore, NLR, PLR, SII, and MLR were investigated as severity markers in RSV infection. However, findings regarding NLR remain inconsistent, as both increased and decreased NLR values were reported to be associated with severe disease across different studies. In contrast, elevated MLR and SII levels showed a more consistent association with disease progression and greater severity of RSV infection. Meanwhile, PLR has generally demonstrated limited prognostic value, with most studies reporting non-significant associations with RSV severity [27,28,29,30,31,32,33,39].

Studies on SARS-CoV-2 have examined the performance of CBC-derived inflammatory markers for the diagnosis and severity assessment of COVID-19. The blood-based markers assessed for diagnostic purposes include NLR, dNLR, LMR, MLR, EMR, ELR, BLR, PLR, MPV/PLT, LYM*PLT, SII, SIRI, AISI, and NLPR. Among these biomarkers, LMR, EMR, ELR, BLR, and LYM*PLT consistently demonstrated significantly lower values in patients diagnosed with COVID-19 across multiple studies. In contrast, SIRI, AISI, and NLPR tended to be significantly elevated in COVID-19 patients. The diagnostic utility of NLR, MLR, PLR, and MPV/PLT remains inconclusive, as their values did not differ substantially between COVID-19-positive patients and control groups, warranting further investigation. NLR, dNLR, MLR, and PLR were also used for evaluating COVID-19 severity. Findings from multiple studies consistently indicate that all of these blood-based markers were increased in the severe COVID-19 group [34,35,36,37,38,39]. Further description of viral studies is available in Table S6. In that table, the viral pathogens are grouped by genome type and envelope, distinguishing the non-enveloped double-stranded DNA adenoviruses from the enveloped negative-sense RNA viruses (metapneumovirus, parainfluenza viruses, influenza, and respiratory syncytial virus) and the enveloped positive-sense RNA SARS-CoV-2.

The performance of CBC-derived inflammatory markers in viral LRTI differed overall from that observed in bacterial LRTI. Among the evaluated markers, LYM*PLT demonstrated the best diagnostic performance. The reported optimal cut-off values ranged from 490.68 to 1742.3, with sensitivity up to 90.67% and specificity as high as 100%. In addition, NLR showed the best prediction, with AUC values exceeding 0.71. Meanwhile, PLR potentially distinguished etiologies with AUC up to 0.779.

NLR was the most frequently studied marker in viral LRTIs, but its findings were less consistent than those observed in bacterial infections. Elevated NLR was reported in severe HMPV, HPIV, RSV, and SARS-CoV-2 infection, whereas no significant association was found in adenovirus pneumonia. Higher PLR was reported in severe adenovirus pneumonia, HMPV infection, and children with HPIV requiring PICU admission, whereas most RSV studies found little or no association with disease severity. Together, these findings suggest that the clinical value of PLR also varies according to the underlying viral pathogen.

LMR was investigated less often than NLR or PLR, but the available studies reported consistent findings. Lower LMR was associated with severe HPIV infection requiring PICU admission and was also useful for identifying influenza B. Most studies of composite inflammatory indices focused on either RSV or SARS-CoV-2 infection. In RSV, lower SII values were associated with diagnosis, whereas higher SII was reported in children with more severe diseases. By comparison, SIRI, AISI, and NLPR were investigated mainly in pediatric COVID-19 and were generally elevated in infected children. Since these markers have rarely been evaluated across a wider range of viral pathogens, it remains unclear whether their clinical value extends beyond these specific infections.

Evidence for the remaining CBC-derived markers was relatively limited. Lower PNR was associated with severe HMPV infection but not adenovirus severity, while dNLR was independently associated with PICU admission in children with HPIV infection. In pediatric COVID-19, EMR, ELR, BLR, and LYM*PLT consistently differed between infected children and controls. Because these markers have been investigated in only a few viral infections, it remains unclear whether they have similar value across other viral LRTIs. Further information is shown in Table 4.

### 2.5. Performance of CBC-Derived Inflammatory Markers in Mixed-Pathogen LRTI

CBC-derived inflammatory markers have also been applied to LRTI caused by mixed pathogens, including combinations of bacterial species, viral species, or both. NLR, PLR, LMR, and SII have been repeatedly used to differentiate bacterial infections from other etiologies. One study used these markers to distinguish empyema from parapneumonic effusion. Across studies, NLR and SII tend to be elevated in bacterial infections, whereas LMR tends to be decreased. However, findings regarding PLR were inconsistent, with some studies reporting increased PLR values and others reporting decreased PLR values in cases of bacterial infection. Increasing SII and AISI have been associated with greater severity of bacterial infection [19,42,43,44,46,47]. A study that examined NLR, NMR, LMR, PLR, EMR, MPVLR, SII, SIRI, and AISI in mixed viral infection found that NLR, NMR, PLR, and MPVLR correlated inversely with the pediatric respiratory severity score (PRESS), with lower values in children with higher scores. The remaining markers showed a non-significant relationship with PRESS [49].

Significantly elevated NLR and LMR were identified as strong indicators of LRTI. In addition, significantly increased PLR, accompanied by decreased MLR and PNR, was observed in pediatric patients hospitalized for more than six days. Furthermore, elevated PLR was associated with the development of neonatal sepsis [45,46,47,48]. Detailed information on the performance of CBC-derived inflammatory biomarkers in mixed-pathogen LRTI is presented in Table S7.

Mixed-pathogen LRTI, caused by concurrent bacterial and viral infections, exhibited variable laboratory findings across the included studies. LMR performed second only to SII in that study, with an optimal cut-off value of 2.26, sensitivity of 75%, and specificity of 69.8%. The findings for LMR were based on relatively few studies. Lower LMR was reported in children with empyema compared with parapneumonic effusion, while another study found higher LMR in children with lower respiratory tract infection.

NLR showed more consistent findings in bacterial-predominant mixed infections than in predominantly viral disease, achieving AUC values up to 0.888. Higher NLR was reported in children with empyema, bacterial pneumonia, and community-acquired pneumonia, and several studies also found it useful for distinguishing bacterial from non-bacterial infections and lower from upper respiratory tract infections. In contrast, studies of predominantly viral mixed infections did not find a significant association between NLR and bronchiolitis severity or respiratory severity scores. Overall, the clinical value of NLR appears to depend on the predominant pathogen involved.

Compared with NLR, the findings for PLR were less consistent. Higher PLR was reported in children with empyema and was also associated with prolonged hospitalization and neonatal sepsis. In contrast, studies of predominantly viral bronchiolitis and mixed viral infections generally did not find a significant relationship between PLR and disease severity. These findings suggest that the usefulness of PLR differs according to the predominant pathogen and the clinical outcome being assessed.

Higher SII was associated with empyema and was independently associated with hospital-acquired otitis media in children with bacterial pneumonia. AISI showed similar findings and was also identified as an independent predictor of this complication. Neither index was significantly associated with respiratory severity scores in mixed viral infection. Overall, the findings suggest that these composite indices have been more useful in bacterial-predominant than predominantly viral mixed infections.

Beyond the commonly studied markers, only a few studies evaluated MLR, PNR, ELR, NPR, LPR, and LNR. Lower MLR and PNR were associated with prolonged hospitalization, while an additional study reported that higher PLR was associated with neonatal sepsis in mixed-pathogen pneumonia. Because these markers have been investigated in only a few studies, their role in mixed-pathogen pediatric LRTIs remains unclear. Further information is provided in Table 5.

### 2.6. Synthesis of Findings

The synthesis below is organized according to four distinct clinical questions with different comparator populations: (i) diagnostic discrimination, in which infected children are compared with healthy or non-infected controls; (ii) etiological differentiation, in which two pathogen groups are compared among symptomatic children; (iii) severity assessment, in which severe and non-severe cases of the same infection are compared; and (iv) outcome prediction, in which markers are related to subsequent clinical events such as intensive care admission. These endpoints are not interchangeable: performance against healthy controls does not imply equivalent performance in the clinically more demanding setting of differential diagnosis among symptomatic children, and thresholds derived for one comparison should not be transferred to another. The comparator population for every quantitative estimate is specified in the Target (comparison) column of Table 3, Table 4 and Table 5, and 95% confidence intervals are provided where the primary studies reported them; AUC values should be interpreted together with the corresponding sensitivity, specificity, sample size, and comparator population. Unless stated otherwise, the findings summarized here derive from retrospective or cross-sectional analyses and denote statistical association or diagnostic discrimination rather than prospectively validated prediction. None of the cut-offs tabulated in this review was accompanied by external validation in an independent pediatric cohort; the one multicenter study that included an external validation cohort [6] reported group differences rather than a validated threshold, and improved performance of combined biomarker models or of models incorporating clinical variables was not attributed to the individual CBC-derived markers they contain.

Age is a pervasive source of heterogeneity in this evidence base rather than a peripheral limitation. Leukocyte differential counts change substantially during childhood, with a physiological lymphocyte predominance between approximately the first week of life and four to five years of age, so an identical ratio does not carry the same meaning in an infant and an adolescent. Two included studies illustrate the consequences: Huang et al. [39] stratified children into four age categories and reported age-dependent diagnostic performance of routine blood parameters, and Zhou and Qian found that SII and SIRI performed poorly in children aged 6 months to 1 year but adequately in children below 6 months and above 1 year [18]. Age-stratified results are indicated in the per-marker tables where the primary studies reported them, and unstratified summaries should be read as averages across physiologically distinct age groups.

Because the leukocyte differential evolves over the course of an infection, a ratio measured at a single time point reflects only one point of a dynamic process. In pertussis, the hematological picture differs between the catarrhal, paroxysmal, and convalescent stages (Section 3.1); in tuberculosis, neutrophil recruitment precedes monocyte recruitment (Section 3.2); and in acute viral infection, early neutrophilia may give way to lymphocyte recovery during convalescence. Reported values should therefore be interpreted as reflecting the specific, usually early, time point at which blood was drawn, and apparent contradictions between studies may partly reflect differences in sampling time relative to symptom onset rather than true differences in marker behavior.

#### 2.6.1. Diagnostic Value of CBC-Derived Inflammatory Markers

Diagnostic performance varied by both marker and pathogen, and no single ratio was consistently informative across all etiologies. NLR exhibited conflicting findings across the majority of single-pathogen infections (*B. pertussis* [5,6], *M. tuberculosis* [8], *M. pneumoniae*, Influenza [11,14], RSV, and SARS-CoV-2) [28,29,34,35,39] yet was consistently elevated in mixed bacterial and mixed pathogen infections [19,41,47]. MLR demonstrated opposing results in *B. pertussis*, *M. pneumoniae*, and SARS-CoV-2; it was not significant in *M. tuberculosis* and was uninvestigated in other groups. PLR showed a significant reduction in *B. pertussis*, inconsistent levels in *M. pneumoniae*, RSV, SARS-CoV-2, and mixed bacterial infections, and non-significant differences in *M. tuberculosis*. NMLR and SII were consistently elevated in specific subsets, with NMLR elevated in *B. pertussis* and *M. tuberculosis*, and SII elevated in *M. pneumoniae*, SARS-CoV-2, and mixed infection groups, despite conflicting results in *B. pertussis* and RSV. SIRI was elevated in SARS-CoV-2 but yielded non-significant or conflicting findings in *B. pertussis*, *M. pneumoniae*, and RSV. Conversely, LMR consistently demonstrated a significant reduction in SARS-CoV-2, mixed bacterial, and mixed pathogen infections, though it yielded conflicting values in Influenza and non-significant results in RSV. Among the remaining markers, MPV/PLT and LYM*PLT displayed conflicting results in Influenza and non-significant associations in RSV, whereas LYM*PLT was significantly reduced in SARS-CoV-2. Finally, novel systemic inflammatory indices (dNLR, AISI, and NLPR) were uniformly elevated in SARS-CoV-2, whereas EMR, ELR, and BLR were consistently reduced in SARS-CoV-2, though these six markers remain largely uninvestigated across other pediatric LRTI etiologies. The pattern that emerges is that consistency depends on whether a single cell population drives the ratio. PLR in pertussis and NMLR in tuberculosis behaved predictably, plausibly because one mechanism dominates, namely toxin-mediated lymphocytosis and sequential neutrophil then monocyte recruitment, respectively. By contrast, MLR and SIRI were discordant precisely where numerator and denominator move together, as in *Mycoplasma pneumoniae* infection, in which NF-κB-driven apoptosis is proposed to deplete monocytes and lymphocytes at rates that may differ between cohorts; this mechanism is biologically plausible but has not been directly demonstrated in the included clinical studies, and differences in disease stage, age distribution, and comparator selection across studies are likely additional contributors (Table S1).

#### 2.6.2. Etiological Differentiation Using CBC-Derived Inflammatory Markers

CBC-derived inflammatory markers have also been assessed for their ability to distinguish between etiologies of pediatric LRTI. The markers evaluated across comparative pathogen groups were NLR, PLR, SII, and SIRI. NLR consistently exhibited significantly higher values in *Mycoplasma pneumoniae* (MP) compared with viral infections, as well as in mixed bacterial infections compared with other pathogen infections. PLR yielded conflicting findings when differentiating between *Mycoplasma pneumoniae* and viral etiologies and remained uninvestigated in mixed bacterial versus other pathogen comparisons. Both SII and SIRI distinguished atypical bacterial from viral causes, consistently showing significant elevation in *Mycoplasma pneumoniae* cases and corresponding significant reductions in viral infections; however, neither marker was evaluated in the context of mixed bacterial versus other pathogen infections. The separation of bacterial from viral disease therefore rests on divergent behavior of the two cell lines that most CBC ratios contain. In *Mycoplasma pneumoniae* infection, neutrophils are recruited rapidly and are not subject to NF-κB-induced apoptosis, whereas lymphocytes are, which may explain why neutrophil-based ratios rise while lymphocyte-based ratios fall. Where a viral infection produces the same directional change, as in SARS-CoV-2, these ratios lose discriminatory value, which is consistent with the conflicting results reported for PLR (Table S2) [17,18,20,43,44].

#### 2.6.3. Severity Assessment Using CBC-Derived Inflammatory Markers

Severity assessment was the most frequently studied application of these markers. NLR was consistently elevated in severe cases of *Bordetella pertussis* [4], *Mycobacterium tuberculosis* [9], *Mycoplasma pneumoniae* [13,15], *Streptococcus pneumoniae* [21], and SARS-CoV-2. However, it showed conflicting findings in HMPV, RSV, and mixed pathogen infections, non-significant differences in HAdV [24,25,27,30,31,32,33,36,37,38], and a significant reduction in mixed viral infections. NEUT% was significantly elevated in severe HAdV [22,23,42,45,48,49] cases. MLR was elevated in severe *M. pneumoniae* infections, but was significantly reduced in mixed viral infections, yielded non-significant findings in RSV and mixed pathogen cases, and was uninvestigated in others. PLR demonstrated consistent elevation across severe *M. pneumoniae*, *S. pneumoniae*, HAdV, and HMPV cases, but showed conflicting findings in RSV, non-significant associations in mixed pathogens, and a significant reduction in mixed viral infections. PNR displayed variable utility, showing a significant reduction in *S. pneumoniae*, elevation in HMPV, non-significant results in HAdV, and conflicting findings in mixed pathogen infections. SII and SIRI were both significantly elevated in severe *M. pneumoniae*, with SII also showing consistent elevation in severe RSV and mixed bacterial infections, though both indices yielded non-significant findings in mixed pathogen and mixed viral cases. LMR, NPR, LPR, and LNR yielded non-significant findings in both mixed pathogen and mixed viral infections. Among other novel indices, MPVLR was significantly reduced in severe mixed viral infections, whereas dNLR was consistently elevated in severe RSV and SARS-CoV-2 cases, and AISI was significantly elevated in mixed bacterial infections. Finally, EMR yielded non-significant results in mixed viral cases, while ELR was investigated in mixed pathogen infections without reaching statistical significance. NLR was most reliable in bacterial disease, where severity is accompanied by sustained neutrophil recruitment into the circulation. Its inconsistency in RSV may be explicable on the same grounds: granulocytes are recruited into the infected airway rather than retained in blood, so the circulating neutrophil count, and therefore NLR, may fall even as disease worsens. Sampling time relative to symptom onset is likely to be a further source of discordance, as it is across the stages of pertussis. Differences in age distribution, comparator selection, and severity definitions between cohorts are further plausible contributors to the discordant directions reported for NLR, MLR, and SIRI across pathogens and settings. Most severity analyses dichotomized cohorts into severe versus non-severe disease; whether these ratios rise proportionally with increasing severity, or merely discriminate severe from non-severe disease, cannot be determined from the current evidence, with the exception of analyses relating markers to graded measures, such as the reported increase in NLR with increasing tuberculosis severity [9] and the correlation of several ratios with the pediatric respiratory severity score in mixed viral infection [49] (Table S3).

#### 2.6.4. Outcome Prediction of CBC-Derived Inflammatory Markers

Fewer studies have examined outcome prediction, and all of these were in viral LRTI. Twelve markers were assessed across three cohorts. In human parainfluenza virus infection, NLR, dNLR, and PLR were significantly elevated, and LMR significantly reduced in children requiring intensive care admission. In RSV infection, NLR and MLR discriminated between children who required intensive care admission and those who did not. By contrast, in a cohort of viral bronchiolitis in which RSV predominated, none of the eleven markers examined, including NLR, PLR, MLR, LMR, SII, and SIRI, were significantly associated with the bronchiolitis risk-of-admission score. Outcome prediction is the weakest part of this evidence base. It rests on a few viral cohorts with heterogeneous endpoints, ranging from admission to the pediatric intensive care unit to risk of hospitalization for bronchiolitis, and no bacterial or mycobacterial infection has been examined for this purpose. Marker performance therefore appears to depend on which outcome is being predicted rather than on the marker itself (Table S4) [26,30].

## 3. Immunopathogenesis of Bacterial LRTI Underlying CBC-Derived Inflammatory Markers

In Section 3 and Section 4, mechanisms that are established in the immunological literature are distinguished from mechanistic explanations proposed for the observed CBC-derived ratios. Where a mechanism has not been directly investigated in the cited pediatric cohorts, it is presented as a hypothesis (for example, “may explain” or “is consistent with”) rather than as a demonstrated finding.

### 3.1. Bordetella pertussis

*Bordetella pertussis* (BP) causes pertussis, a highly contagious acute respiratory infection that progresses through three clinical stages [50]. The catarrhal stage presents with nonspecific symptoms and may be indistinguishable from other respiratory infections. The paroxysmal stage follows, defined by spasmodic coughing episodes with the classic “whoop”. The convalescent stage involves a gradual decline in cough severity as bacterial clearance proceeds. This staging matters for marker interpretation, because the hematological picture differs markedly between stages [51].

BP enters the airway via droplets, binds to ciliated epithelial cells, and colonizes the nasopharynx. It produces several toxins, of which pertussis toxin is the most relevant to the blood count. Pertussis toxin blocks the ability of lymphocytes to enter tissues, which causes a marked increase in circulating lymphocytes. During the catarrhal stage, the innate response predominates, with macrophages, dendritic cells, and neutrophils responding to bacterial components. The transition to adaptive immunity occurs during the paroxysmal stage and is marked by the surge in circulating lymphocytes [51,52,53].

In pertussis, NLR, MLR, and NMLR are increased when used as diagnostic and severity markers, whereas PLR is decreased across all studies. These markers are all calculated with the lymphocyte count as the denominator. Despite the high number of lymphocytes, neutrophils and monocytes gradually rise as the need for innate immunity also rises. This pattern may explain the observed increases in NLR, MLR, and NMLR. Platelets are acute-phase reactants that increase during inflammation. Platelet count in pertussis may remain unchanged or increase slightly. Due to high lymphocyte counts as the denominator, PLR decreases significantly. SIRI, on the other hand, is inconsistently reported, with some studies showing it is significantly increased and others showing no significant change. SIRI may reflect the shift between the catarrhal stage and the paroxysmal stage. This variability is likely due to the timing at which the sample is taken. Samples taken earlier, during the catarrhal stage, may show a lower ratio, while samples taken during the convalescent stage may show an increase in the ratio. Combined use of these markers could contribute to diagnosing the disease, as well as measuring the severity of the disease [54,55,56].

### 3.2. Mycobacterium tuberculosis

Tuberculosis (TB), the leading cause of death from a single infectious agent, is caused by *Mycobacterium tuberculosis* (MTB) [57]. It primarily affects the lower respiratory system, with chronic cough, low-grade fever, night sweats, and weight loss [58]. MTB is a thin, slightly curved, acid-fast bacillus that stains poorly by the Gram method, and its cell wall contains waxes and complex glycolipids [59]. Only about 10% of infected people develop disease; the remainder maintain latent infection, in which MTB lies dormant in the lungs [58].

Airflow and lung anatomy favor deposition of MTB in the alveoli, where alveolar macrophages engulf the bacilli. Bacilli that evade destruction persist within macrophages. These infected macrophages release inflammatory cytokines (TNF-α, IL-1β, IFN-γ) and chemokines (CXCL8, CCL2, CXCL10), which recruit dendritic cells, neutrophils, T lymphocytes, and monocytes to the site of infection. Innate and adaptive immunity then combine to isolate MTB within a granuloma. This occurs in at least 90% of infected subjects and sequesters lymphocytes in tissue [60,61,62,63].

Significant hematological markers in TB are NLR, MLR, and NMLR. During disease progression, neutrophils and monocytes are actively recruited by IL-8 and CCL2, respectively, which increases NLR and MLR. NMLR may reflect the shift between neutrophil recruitment and monocyte recruitment, as neutrophil numbers increase from minutes to hours after infection while monocyte numbers increase hours to days after infection. The number of circulating lymphocytes may fall as most migrate to tissues, leading to higher NLR, MLR, and NMLR. Meanwhile, PLR increases variably with disease severity [64,65,66,67].

### 3.3. Mycoplasma pneumoniae

*Mycoplasma pneumoniae* (MP) accounts for about 20 to 40% of community-acquired pneumonia (CAP) in children [68]. CAP ranges from mild disease to severe life-threatening conditions, with fever, chills, cough, purulent sputum, dyspnea, and pleuritic chest pain [69,70]. MP also causes extrapulmonary disease affecting the skin, kidneys, gastrointestinal tract, heart, musculoskeletal system, brain, and hematological system [71].

MP attaches to the surface of the bronchial ciliary epithelium and causes direct damage through nutrient depletion, cytoskeletal rearrangement, toxin production, oxidative damage, and induction of apoptosis [72,73,74].

The mechanism most relevant to the blood count is immune-mediated. Lung macrophages recognize MP through TLR2 and activate the MyD88-NF-κB signaling pathway. Activation of NF-κB causes severe inflammation and induces apoptosis of monocytes, macrophages, and lymphocytes, ultimately reducing immune function. Pro-inflammatory cytokines including IFN-γ, TNF-α, and multiple interleukins are released. Neutrophils accumulate rapidly at the site of infection through chemokine-mediated chemotaxis. Critically, neutrophils are not subject to NF-κB-induced apoptosis, which distinguishes their behavior from that of monocytes and lymphocytes [75,76,77,78,79].

In MP infection, neutrophils are highly increased in the early immune response, leading to increased NLR. MLR can increase, decrease, or remain unchanged depending on the rate of apoptosis induced by the NF-κB pathway. This may account for the discrepancy between the findings of Feng et al. [11] and those of Shao et al. [14]. MLR falls when monocyte apoptosis outpaces lymphocyte apoptosis and rises when the reverse occurs. SII reflects high neutrophils and platelets with low levels of lymphocytes. This is consistent with lymphocyte apoptosis alongside relatively preserved neutrophil and platelet counts, since neutrophils are not affected by NF-κB pathway. SIRI values vary between studies, reflecting differences in the rate of lymphocyte and monocyte apoptosis. Significant markers in MP infection, such as NLR and SII, which show consistent results, may be effectively applied to diagnosing MP, determining its severity, and differentiating MP infection from other pneumonias [80,81,82,83].

### 3.4. Streptococcus pneumoniae

*Streptococcus pneumoniae* (SP) is a Gram-positive diplococcus [84]. SP is normal flora of the respiratory tract and is usually carried asymptomatically. In immunocompromised conditions, it causes opportunistic infections including pneumonia, otitis media, and meningitis [85,86].

Transmission occurs through aerosol droplets and close contact. Colonization is considered necessary for disease, although many colonized individuals remain asymptomatic [87,88,89].

Pneumococcal pneumonia is characterized by pulmonary inflammation resulting from bacterial factors that trigger pro-inflammatory cytokine responses and the recruitment of immune cells. SP simultaneously employs strategies to evade that response. The result is a vigorous but partly ineffective inflammatory response, with sustained cytokine-driven recruitment of neutrophils [90,91,92,93].

The severity of pneumococcal disease is due to a significant inflammatory response induced by the activation of complement pathways and cytokine release. NLR, PLR, and PNR are useful determinants of disease severity. NLR increases in response to a significant increase in neutrophils due to pro-inflammatory cytokines that recruit immune cells. The increase in PLR may be explained by an increase in platelet count. There is also a decrease in PNR since neutrophils increase. While platelets also rise, the increase in neutrophils is often greater, leading to a decrease in PNR. Therefore, increased NLR and PLR and decreased PNR are consistent with the inflammatory response and immune dysregulation that characterize severe SP infection [94,95,96,97].

## 4. Immunopathogenesis of Viral LRTI Underlying CBC-Derived Inflammatory Markers

### 4.1. Human Adenovirus

Human adenoviruses (HAdV) can cause infection at any age but most commonly in children and infants; by 10 years of age, most children have had at least one episode [98]. Most infections are asymptomatic. Symptoms resemble the common cold, including rhinorrhea, fever, cough, and sore throat, but lower respiratory infections such as bronchitis, bronchiolitis, and pneumonia can be severe and even fatal [22].

HAdV are medium-sized (70 to 100 nm), non-enveloped viruses with an icosahedral nucleocapsid containing a double-stranded linear DNA genome [98,99]. HAdV binds cell surface receptors and triggers internalization by endocytosis [100,101].

Infection triggers a strong innate response. Type I interferon, specifically IFN-α and IFN-β, is produced by infected cells and inhibits viral replication. TNF-α, rapidly produced by macrophages and dendritic cells, stimulates a systemic acute-phase response, induces C-X-C chemokine generation in type II epithelial cells, and induces colony-stimulating factor expression in vascular endothelial cells. This activation leads to neutrophil chemoattraction and release of additional inflammatory factors [102,103,104].

Significant markers in HAdV infections include NEUT% and PLR. The virus activates NF-κB, leading to the production of cytokines and the release of neutrophils from the bone marrow into the bloodstream. There is thrombocytopenia with an even more pronounced loss of lymphocytes, and the rise in PLR is consistent with a fall in lymphocytes that exceeds the fall in platelets. By contrast, NLR and PNR did not differ significantly by severity in the included studies: although neutrophilia with lymphopenia would be expected to raise NLR, and thrombocytopenia with neutrophilia to lower PNR, neither pattern reached statistical significance, illustrating that mechanistically plausible changes are not always clinically discriminative [98,105,106].

### 4.2. Human Metapneumovirus

Human metapneumovirus (HMPV) was identified in 2001 and is a common cause of respiratory tract infection in children and adults, and of severe disease in immunocompromised hosts [107]. It is found predominantly in children under 2 years of age, with an average age of 22 months [108]. Seroprevalence studies indicate that approximately 90 to 100% of children are infected by 5 to 10 years of age [109].

HMPV, measuring 150 to 600 nm, is an enveloped virus with a single-stranded, non-segmented, negative-sense RNA genome that encodes nine proteins [110]. The G and F glycoproteins are the main functional molecules for adhesion to and infection of target cells: G serves as the primary surface structural glycoprotein and is crucial for viral adhesion, while F mediates fusion between the viral envelope and the cell membrane, establishing infectivity. The G protein also shapes the immune response in ways that bear on circulating cell counts. It mediates both innate and adaptive responses involving neutrophils, dendritic cells, natural killer (NK) cells, and B cells [111,112,113,114].

Neutrophils rise along with disease severity, so an elevated NLR is associated with severe outcomes and acts as a marker of a complex inflammatory state. HMPV can directly impair the function of T cells. Studies have shown that the virus can trigger an exhausted state in CD8+ T cells, reducing their ability to produce IFN-γ, leading to lymphopenia in HMPV infection. This may further account for the observed increase in NLR. PLR is also increased, and the same mechanism may be involved. On the other hand, PNR decreases as neutrophil counts rise relative to platelet counts [115,116,117,118].

### 4.3. Human Parainfluenza Viruses

Human parainfluenza viruses (HPIVs) are a common cause of acute respiratory illness throughout life, with infants, children, and the immunocompromised most likely to develop severe disease [119]. They are single-stranded, enveloped RNA viruses of the *Paramyxoviridae* family [120]. Four major serotypes are recognized [121,122].

Infection begins in the nose and oropharynx and then spreads to the lower airways, with peak replication 2 to 5 days after initial infection [123,124,125].

HPIV infection of the airway epithelium causes extensive changes in cellular gene expression and stimulates increased production of numerous cytokines and chemokines that attract and activate cells mediating the immune response. The NF-κB, IRF3, and type I IFN pathways play a major role in regulating this antiviral and inflammatory response [126,127].

The increase in NLR, dNLR, and PLR, and the decrease in LMR are driven by the following immunological events during HPIV infection: neutrophilia, lymphopenia, monocyte response, and platelet changes. Neutrophilia and lymphopenia drive the NLR and dNLR higher. While monocytes are recruited to the site of infection, a decreased LMR indicates that the fall in lymphocytes outweighs any change in monocyte count. An elevated PLR suggests that platelet counts are relatively high compared with the lymphocyte count [128,129].

### 4.4. Influenza

Influenza is an acute respiratory infection caused by influenza viruses. In 2018, an estimated 109.5 million influenza virus episodes occurred in children under 5 years, with up to 34,800 influenza virus-associated deaths [130]. Symptoms usually begin 2 days after infection and include sudden onset of fever, dry cough, headache, muscle and joint pain, severe malaise, sore throat, and runny nose. Patients commonly recover within 1 week, although influenza can cause severe disease in people at high risk [131,132].

There are four types of influenza virus. Seasonal influenza A and B circulate among humans worldwide, and only type A is able to cause pandemics. Influenza A is classified into subtypes according to the combination of surface proteins, currently A (H1N1) and A(H3N2). Influenza B is divided into the B/Yamagata and B/Victoria lineages [133,134].

Host pattern recognition receptors activate the transcription factors IRF3, IRF7, and NF-κB, causing the expression of a variety of interferons and cytokines and increasing the numbers of dendritic cells, macrophages, neutrophils, monocytes, and NK cells [130,135,136,137].

Huang et al. [39] reported that NLR, LMR, MPV/PLT, and LYM*PLT in influenza did not differ significantly. Compared with Zhu et al. [40], the study showed an increase in NLR and MPV/PLT, along with a decrease in LMR and LYM*PLT. This is due to the difference in study designs. Huang et al. [39] focused on differentiating similar respiratory infections in children between SARS-CoV-2, influenza A, and RSV. The results showed no significant differences between the three infections. However, when healthy and infected groups were compared in the study by Huang et al. [39], the results were consistent with those of Zhu et al. [40]. By contrast, Zhu et al. [40] focused on differentiating influenza A from influenza B. Results showed a significant increase in NLR and MPV/PLT when influenza B patients were compared with healthy individuals, with no significant difference between influenza A and influenza B patients. This indicates that these markers may be useful for distinguishing infected patients from healthy individuals [39,40].

### 4.5. Respiratory Syncytial Virus

Respiratory syncytial virus (RSV) represents the leading global cause of acute respiratory infection [138]. RSV infects approximately 68% of children within the first years of life, with recurrent infections throughout adulthood due to limited durable immunity [139]. The annual worldwide burden has been estimated at roughly 3.6 million hospitalizations and about 100,000 deaths in children younger than 5 years [140].

RSV, a non-segmented, negative-sense, single-stranded RNA virus, belongs to the genus *Orthopneumovirus* within the family *Pneumoviridae* [141]. It spreads through respiratory droplets and follows an incubation period of 2 to 8 days [142]. After inoculation through the nasopharynx or conjunctiva, the virus targets ciliated epithelial cells within the respiratory tract [143].

Most of the damage to the airway is mediated by the immune response rather than by viral replication itself. Cell-derived IL-33 induces the release of NF-κB and IRF from alveolar macrophages and mast cells during the innate response, resulting in the production of IL-6 and TNF-α and secretion of tissue mucus. Accumulation of these inflammatory factors recruits granulocytes, particularly neutrophils, to the infected site. IL-33 also increases the expression of thymic stromal lymphopoietin in dendritic cells, shifting T cell differentiation towards CD4+ T cells expressing Th2 cytokines [53,144,145].

Pan et al. [29] reported low NLR in RSV, which may be explained by continuous neutrophil migration into the infected sites, lowering the circulating neutrophil count, while the lymphocyte count rises with the CD4+ cell response. PLR and SII were also lower in RSV [28]. LMR and LYM*PLT were also significantly lower in RSV patients, indicating their use for diagnostic purposes. MPV/PLT values were significantly higher in RSV than in healthy cohorts. Incorporation of these markers could provide useful parameters for diagnosing RSV [28,29].

### 4.6. SARS-CoV-2

The emergence of severe acute respiratory syndrome coronavirus 2 (SARS-CoV-2) has caused a global pandemic [146]. Coronaviruses are enveloped, positive-sense, single-stranded RNA viruses of the family *Coronaviridae*, which produce mild to severe respiratory disease in humans [147]. COVID-19 shows a complex profile with many different clinical presentations: patients may be asymptomatic or experience mild, moderate, or severe symptoms, and may present with or without pneumonia [148]. Hypoxia is the common feature and, if it worsens, may lead to acute respiratory distress syndrome (ARDS) [149].

The spike (S) protein is the primary mediator of SARS-CoV-2 binding to the host–cell receptor ACE2 and of subsequent membrane fusion, with entry into epithelial cells facilitated by transmembrane serine protease 2 (TMPRSS2) [150,151,152].

SARS-CoV-2 damages type-2 alveolar cells, which are essential for surfactant synthesis and repair of damaged tissues. The viral genome and proteins act as a pathogen-associated molecular pattern and stimulate the innate immune system, leading to production of type I interferon and a broad range of other cytokines. Monocytes release pro-inflammatory cytokines responsible for pneumocyte apoptosis, and macrophages release chemokines and cytokines that increase capillary permeability and recruit neutrophils. Excessive degranulation of neutrophils causes permanent damage to pneumocytes, breaking the alveolar-capillary barrier. CD8+ cytotoxic T cells lyse virus-infected cells, but lymphopenia has been reported to correlate with disease severity and mortality [153,154,155].

Neutrophil-driven markers (NLR, dNLR, SII, SIRI, AISI, NLPR) are all increased, consistent with the neutrophilia coupled with lymphopenia that characterizes COVID-19. Thrombocytopenia along with lymphopenia further drives markers such as SII, AISI, and NLPR. The increase in MLR is consistent with the monocyte response to SARS-CoV-2. EMR is decreased because of eosinopenia. LMR, LYM*PLT, and MPV/PLT show a decrease because of lymphopenia, monocytosis, and thrombocytopenia. ELR and BLR are decreased as a direct consequence of eosinopenia and basopenia [34,35,156,157,158].

## 5. Limitations

Several limitations should be considered when interpreting this review. The evidence base is dominated by retrospective, single-center designs (36 of 46 studies), and is geographically concentrated, with 38 of 46 studies from China and Turkey, so selection and information bias cannot be excluded and the reported thresholds may not transfer to other populations, including Southeast Asian children. The included studies differ in age structure, comparator populations, timing of blood sampling, and definitions of severe disease, which range from radiological criteria to intensive care admission; an elevated ratio described as predicting severity therefore does not denote the same clinical state in every cohort. Almost no study reported age-stratified thresholds, although neutrophil and lymphocyte proportions change substantially over the first years of life, and the timing of sampling relative to symptom onset was rarely stated. The cut-offs reported here are not tabulated by pathogen, age group, endpoint, comparator population, or study design, and no threshold should be transferred from one of these settings to another; the ranges quoted in the text describe the spread of published values rather than a usable clinical interval. Cut-offs were also generally identified in the same dataset in which their performance was estimated, with internal or external validation rarely reported, so the accompanying estimates are likely to be optimistic; comparisons against healthy controls further overstate accuracy relative to the clinical task of discriminating among symptomatic children. Finally, the narrative design precludes pooled estimates and formal risk-of-bias appraisal, no structured instrument was applied, and the search was restricted to a single database and to English-language full-text articles, so eligible studies may have been missed and studies reporting significant performance may be over-represented.

## 6. Conclusions

This review synthesized 46 studies of CBC-derived inflammatory markers in pediatric LRTI and mapped their performance across diagnosis, etiological differentiation, severity, and outcome prediction. NLR was the most frequently investigated marker and showed the broadest evidence base for severity assessment and for distinguishing bacterial from viral disease, although its performance remained pathogen-, population-, and endpoint-dependent, and no single ratio performed consistently across all etiologies. The principal contribution of this review is to relate that inconsistency to pathogen biology: ratios governed by a single dominant mechanism, such as PLR in pertussis and NMLR in tuberculosis, behaved predictably, whereas ratios whose numerator and denominator respond to the same process, such as MLR and SIRI in *Mycoplasma pneumoniae* infection, did not. Three limitations should direct future work: cut-offs were neither standardized nor age-specific; definitions of severe disease differed across studies; and the timing of sampling relative to symptom onset was rarely reported. Evidence for outcome prediction was the most limited of the four applications. Prospective, multicenter studies with age-stratified reference intervals, uniform severity definitions, and pre-specified thresholds validated in independent cohorts are therefore required. Until such data are available, CBC-derived ratios should be interpreted alongside clinical assessment rather than used alone.

## Figures and Tables

**Table 1:** Evaluated CBC-derived inflammatory markers across studies.

Abbreviation	Definition and Formula
NLR	neutrophil-to-lymphocyte ratio (neutrophil count/lymphocyte count)
dNLR	derived neutrophil-to-lymphocyte ratio (neutrophil count/[total leukocytes–neutrophil count])
NPR	neutrophil-to-platelet ratio (neutrophil count/platelet count)
NMR	neutrophil-to-monocyte ratio (neutrophil count/monocyte count)
MLR	monocyte-to-lymphocyte ratio (monocyte count/lymphocyte count)
LMR	lymphocyte-to-monocyte ratio (lymphocyte count/monocyte count)
LPR	lymphocyte-to-platelet ratio (lymphocyte count/platelet count)
LNR	lymphocyte-to-neutrophil ratio (lymphocyte count/neutrophil count)
EMR	eosinophil-to-monocyte ratio (eosinophil count/monocyte count)
ELR	eosinophil-to-lymphocyte ratio (eosinophil count/lymphocyte count)
PLR	platelet-to-lymphocyte ratio (platelet count/lymphocyte count)
PNR	platelet-to-neutrophil ratio (PNR = platelet count/neutrophil count)
BLR	basophil-to-lymphocyte ratio (basophil count/lymphocyte count)
NMLR	neutrophil-monocyte-to-lymphocyte ratio ([neutrophil count + monocyte count]/lymphocyte count)
NLPR	neutrophil-to-lymphocyte-platelet ratio (neutrophil count/[lymphocyte count x platelet count])
MPV/PLT	mean platelet volume-to-platelet count (mean platelet volume/platelet count)
MPVLR	mean platelet volume-to-lymphocyte ratio (mean platelet volume/lymphocyte count)
LYM*PLT	lymphocyte multiplied by platelet (lymphocyte count x platelet count)
SII	systemic immune-inflammation index ([neutrophil count x platelet count]/lymphocyte count)
SIRI	systemic inflammatory response index ([neutrophil count x monocyte count]/lymphocyte count)
AISI	aggregate index of systemic inflammation ([neutrophil count x monocyte count x platelet count]/lymphocyte count)
NEUT%	neutrophil percentage ([neutrophil count/total leukocytes] × 100%)

Marker types: NEUT% is a proportional parameter rather than a ratio; NLR, dNLR, NPR, NMR, MLR, LMR, LPR, LNR, EMR, ELR, PLR, PNR, BLR, MPV/PLT and MPVLR are simple ratios of two cell-count or volume parameters; and NMLR, NLPR, LYM*PLT, SII, SIRI and AISI are composite indices combining three or more parameters. NPR and PNR are reciprocals of one another, as are MLR and LMR and as are LNR and NLR; each is reported here in the direction used by the primary study, and the reciprocal forms are not interchangeable. Ratios of absolute counts are dimensionless and independent of the reporting unit, but MPV/PLT, MPVLR and LYM*PLT combine parameters with different units (mean platelet volume in fL; platelet and lymphocyte counts per litre, per microlitre, or in 10^9/L), and their numerical values therefore depend on the units used by each primary study; the cut-offs quoted for these three markers are reported as published and are not comparable across studies. NMLR and the composite indices are defined on absolute counts; where a primary study did not state whether absolute counts or percentages were used, the value is reported as published.

**Table 2:** Characteristics of studies investigating CBC-derived inflammatory markers in pediatric LRTI.

Study	Country	Study Design	Subject Characteristics	Comorbidities	Etiology	Markers Studied	Key Takeaway
Li C., et al., 2026 [4]	China	Retrospective cohort	A total of 219 children aged 0–14 years with confirmed pertussis: severe (*n* = 21) vs. non-severe (*n* = 198) were observed.	Excluded: severe cardiopulmonary dysfunction, structural airway abnormalities, tuberculosis co-infections.	*Bordetella pertussis*	NLR ^25^	NLR ^25^ was an independent predictor of severe pertussis.
Tang J., et al., 2025 [5]	China	Retrospective observational	CBC ^1^-derived inflammatory markers in children under 18 years old were analyzed among: pertussis group (*n* = 892), non-pertussis group with respiratory tract infection (*n* = 903), and healthy group (*n* = 1.165).	Not restricted.		NLR ^25^, MLR ^26^, PLR ^27^, NMLR ^28^, SII ^29^, SIRI ^30^	NLR ^25^ and NMLR ^28^ demonstrated the highest diagnosis utility for pertussis.
Zhou J., et al., 2025 [6]	China	Retrospective multicenter observational	A total of 1.139 children under 14 years old with suspected pertussis: derivation cohort from first hospital (*n* = 545; 381 in training group vs. 164 in validation group) and external validation cohort from other hospital (*n* = 594). PCR ^2^ test and complete blood count were conducted to confirm pertussis diagnosis.	Not restricted.		NLR ^25^, MLR ^26^, PLR ^27^, SII ^29^, SIRI ^30^	NLR ^25^, PLR ^27^, MLR ^26^, and SII ^29^ provided additional value for the identification of pertussis infection.
Choudhary R., et al., 2019 [7]	Kenya	Longitudinal cohort	A total of 160 children under 12 years old with HIV ^4^ infection differentiated into several groups based on microbiologic confirmation testing: TB ^5^-confirmed (*n* = 13), TB ^5^-unconfirmed (*n* = 67), TB ^5^ unlikely (*n* = 80). The MLR ^26^ value being evaluated for distinguishing confirmed TB ^5^ from other groups among HIV ^4^-infected children.	Excluded: central nervous system infections.	*Mycobacterium* *tuberculosis*	MLR ^26^	MLR ^26^ offered added diagnostic value in identifying HIV ^4^-positive TB ^5^ infection.
Kissling M., et al., 2023 [8]	Switzerland	Prospective multicenter observational	A total of 389 children aged 0–18 years were evaluated: TB ^5^ disease (*n* = 25), TB ^5^ infection (*n* = 12), non-TB ^5^ with LRTI ^6^ (*n* = 324), healthy control (*n* = 28).	Not restricted.		NLR ^25^, MLR ^26^, NMLR ^28^	NLR ^25^ and NMLR ^28^ significantly associated with pediatric TB ^5^-infection.
Ma W., et al., 2024 [9]	China	Retrospective case–control	A total of 235 children aged 0–18 years were identified and grouped based on T-lymphocyte subsets: TB ^5^ group (*n* = 176; 60 had non-severe TB ^5^ vs. 116 had severe TB ^5^), and bacterial CAP ^7^ (*n* = 59).	Excluded: other respiratory infections, HIV ^4^ infection, chronic kidney disease, immunodeficiency, hematological disorders, rheumatic immune diseases, and currently using immunosuppressive therapy.		NLR ^25^	NLR ^25^ was a reliable predictor of TB ^5^ severity.
Rees C., et al., 2020 [10]	Tanzania	Prospective cohort	A total of 239 adolescents aged 13–15 years were screened IGRA ^8^ at day 0 (visit 1), day 60 (visit 2), and day 120 (visit 3). Participants were grouped by IGRA ^8^ results: IGRA ^8^-negative (*n* = 98), all converters (*n* = 47), early converters (*n* = 18), late converters (*n* = 29), transient converters (*n* = 25), and persistent converters (*n* = 22).	Excluded: IGRA ^8^-positive result on first screening (day 0).		NLR ^25^, MLR ^26^, PLR ^27^	All markers did not reflect the development of TB ^5^ infection.
Feng M., et al., 2026 [11]	China	Retrospective observational	A total of 132 hospitalized children with MP ^9^-associated lobar pneumonia underwent bronchoscopy, then grouped based on examination results: PB ^10^ group (*n* = 44) vs. non-PB ^10^ group (*n* = 88).	Excluded: congenital heart disease, arrhythmia, immunodeficiency, severe liver and/or kidney dysfunction, bleeding disorders.	*Mycoplasma pneumoniae*	NLR ^25^, MLR ^26^, PLR ^27^, SII ^29^, SIRI ^30^	PLR ^27^ and SII ^29^ were independent predictors of MPP ^11^-associated plastic bronchitis.
Guo X., et al., 2025 [12]	China	Retrospective cohort	A total of 254 hospitalized children with MPP ^11^: severe group (*n* = 103) vs. non-severe group (*n* = 151).	Excluded: neonatal respiratory distress syndrome, bacterial encephalitis, malignancy, immunodeficiency, genetic disorders.		NLR ^25^, MLR ^26^, PLR ^27^, SII ^29^, SIRI ^30^	All markers potentially used for predicting MP ^9^ severity.
Li D., et al., 2023 [13]	China	Prospective cohort	A total of 1401 patients under 7 years old with MPP ^11^ were monitored up to 60 days prospectively. After follow-up, some patients developed necrotizing pneumonia (*n* = 30), and refractory MPP ^11^ (*n* = 370).	Excluded: disease occurred chronically (>4 weeks), other respiratory disease, congenital heart disease, immunodeficiency, heredity neurological disorder, other pathogen co-infection.		NLR ^25^	NLR ^25^ predicted necrotizing pneumonia and refractory MPP ^11^ as MPP ^11^ complications.
Shao L., et al., 2025 [14]	China	Retrospective case–control	A total of 424 children aged 2–14 years with confirmed positive MP ^9^ DNA ^18^ compared with 150 healthy children.	Excluded: other respiratory infection, severe cardiovascular disease, immune system diseases, and currently using immunosuppressive therapy.		NLR ^25^, MLR ^26^, PLR ^27^, SII ^29^, SIRI ^30^	All markers significantly associated with MP ^9^ infection.
Wang S., et al., 2024 [15]	China	Retrospective cohort	A total of 304 hospitalized children with MPP ^11^: severe group (*n* = 78) vs. non-severe group (*n* = 226).	Excluded: congenital heart disease, genetic disorders, metabolic disease, immunodeficiency, hematological tumor disease, neurological dysplasia, epilepsy, bronchopulmonary dysplasia.		NLR ^25^, PLR ^27^, SII ^29^, SIRI ^30^	SII ^29^ was the strongest inflammatory marker for predicting severe pediatric MPP ^11^.
Zhang K., et al., 2026 [16]	China	Retrospective case–control	A total of 2042 children aged 0–18 years suspected of having MPP ^11^ divided based on PCR ^2^ results: confirmed MP ^9^ infection (*n* = 1637) vs. non-MP ^9^ infection (*n* = 405).			NLR ^25^	NLR ^25^ potentially predicting MPP ^11^.
Chen J., et al., 2024 [17]	China	Retrospective cross-sectional	A total of 423 children under 12 years old diagnosed with MP ^9^ (*n* = 423) and influenza (*n* = 214) were administered routine blood test.	Not restricted	*Mycoplasma pneumoniae* vs. influenza	PLR ^27^	PLR ^27^ as a potent blood-based marker to distinguish MP ^9^ and influenza infection.
Zhou H., Qian K., 2025 [18]	China	Retrospective case–control	A total of 240 children aged between 2 months and 14 years diagnosed with CAP ^7^: RSV ^14^ infection (*n* = 120) vs. MP ^9^ infection (*n* = 120).	Excluded: HAP, chronic lung disease, immunodeficiency, other pathogen co-infection	*Mycoplasma pneumoniae* vs. RSV ^14^	SII ^29^, SIRI ^30^	SII ^29^ and SIRI ^30^ distinguish RSV ^14^ from MP ^9^ CAP ^7^.
Zheng H., et al., 2022 [19]	China	Comparative cross-sectional	This study examined blood tests for patients with bacterial pneumonia (*n* = 50), MPP ^11^ (*n* = 50), and healthy control (*n* = 50). The results were analyzed further among these three groups.	Excluded: hematological malignancy, immunodeficiency, other pathogen infection, prior antibiotic administration, and currently using glucocorticoid therapy.	*Mycoplasma pneumoniae* and bacterial pneumonia	NLR ^25^, PLR ^27^	PLR ^27^ shows the strongest clinical value for differentiating bacterial pneumonia from MPP ^11^ and healthy control.
Wan Y., et al., 2026 [20]	China	Prospective case–control	Participants aged 1–13 years with MPP ^11^ (*n* = 85) and viral pneumonia (*n* = 85) were included in this study.	Excluded: congenital heart disease, immunodeficiency, malignancy, allergy/atopy, using glucocorticoid or immunosuppressant within 4 weeks.	*Mycoplasma pneumoniae* vs. viral pneumonia	NLR ^25^, PLR ^27^	NLR ^25^ significantly distinguished MPP ^11^ from viral pneumonia.
Bao M., et al., 2026 [21]	China	Retrospective cohort	A total of 203 children aged between 28 days and 13 years confirmed with SPP ^12^: severe (*n* = 55) vs. non = severe (*n* = 148).	Excluded: nosocomial pneumonia, immunodeficiency, hematological malignancy, severe congenital disease	*Streptococcus pneumoniae*	NLR ^25^, PLR ^27^, PNR ^31^	All blood-based markers had significant clinical values in predicting SPP ^12^ severity.
Wang N., et al., 2024 [22]	China	Retrospective cohort	A total of 202 children aged between 28 days and 14 years, diagnosed with pneumonia and positive adenovirus PCR ^2^: severe (*n* = 77) vs. non-severe (*n* = 125).	Excluded: multiple injuries, intracranial hemorrhage, required diseases from surgical intervention, and other severe comorbidities not related to pneumonia.	Human adenovirus	NEUT% ^32^	Elevated NEUT% ^32^ reflected severity risk factors.
Xu H., et al., 2025 [23]	China	Retrospective cohort	A total of 428 children under 18 years confirmed positive HAdV ^15^: severe (*n* = 54) vs. non-severe (*n* = 374) adenovirus pneumonia.	Excluded: major comorbidities, including malignancies or nephrotic syndrome.		NLR ^25^, PLR ^27^, PNR ^31^	Only PLR ^27^ that significantly related with HAdV ^15^ severity.
Guo Y., et al., 2025 [24]	China	Retrospective observational	Some HMPV ^13^-positive nasopharyngeal swabs collected from 287 children hospitalized, then being analyzed: severe (*n* = 185) vs. non-severe (*n* = 102) HMPV ^13^ pneumonia.	Not restricted.	Human metapneumovirus	NLR ^25^, PLR ^27^, PNR ^31^	All blood-based markers significantly indicated HMPV ^13^ severity.
Liu L., et al., 2026 [25]	China	Retrospective cohort	A total of 878 pediatrics hospitalized under 17 years old confirmed HMPV ^13^ infection: severe (*n* = 246) vs. mild (*n* = 632) community-acquired pneumonia.	Excluded: positive nosocomial infection.		NLR ^25^	Among HMPV ^13^ infected patients, NLR ^25^ decreased as CAP ^7^ severity increased.
Chu F., et al., 2023 [26]	China	Retrospective cohort	A total of 331 pediatric patients aged between 1 month and 12 years who tested positive for HPIV RNA ^19^, admitted to PICU ^16^ (*n* = 54) and general ward (*n* = 277) underwent laboratory testing.	Excluded: other pathogen infections, hematological disorders, malignancy, and prior chemotherapy.	Human parainfluenza viruses	NLR ^25^, dNLR ^33^, PLR ^27^, LMR ^34^	All markers had predictive performance for assessing clinical outcome.
Okay Z., et al., 2024 [27]	Turkey	Retrospective cohort	Pediatric patients aged 29 days to 2 years who tested positive for RSV ^14^ on a direct fluorescent antibody test divided into four groups: mild, moderate, severe, and healthy. Each group consist of 45 patients.	Not restricted.	RSV ^14^	NLR ^25^	NLR ^25^ was paradoxically lower in disease compared with healthy control.
Okuyan O., et al., 2023 [28]	Turkey	Retrospective case–control	Data collected from 286 children aged 0 to 12 years. RSV ^14^ antigen was detected using a chromatographic immunoassay of nasopharyngeal swab samples. Among 286 children, 138 were RSV ^14^ (+), and 148 were RSV^14^ (−) as a control group.	Excluded: prior recurrent wheezing, recurrent RSV ^14^ infections, other pathogen infections, metabolic disorders, immunodeficiency.		NLR ^25^, PLR ^27^, SII ^29^	Decreased NLR ^25^, PLR ^27^, and SII ^29^ clinically related with RSV ^14^ diagnosis.
Pan J., et al., 2026 [29]	China	Retrospective cohort	A total of 1269 children identified RSV ^14^ pneumonia: severe (*n* = 836) vs. non-severe (*n* = 433).	Excluded: nosocomial infection, hematological malignancy.		NLR ^25^	Elevated NLR ^25^ was identified as a critical predictor of severe RSV ^14^ pneumonia.
Burrack N., et al., 2023 [30]	Southern Israel	Retrospective cohort	A total of 2038 children, aged under 2 years, were diagnosed with RSV ^14^ bronchiolitis; 104 of them were being admitted to the PICU ^16^.	Excluded: congenital heart disease, genetic and chromosomal abnormalities, neuromuscular impairments.		NLR ^25^, MLR ^26^	Higher NLR ^25^ and MLR ^26^ were identified as a risk for severe outcomes, specifically PICU ^16^ admission, among children with RSV ^14^ bronchiolitis.
Li W., et al., 2026 [31]	China	Single-center retrospective cohort	A total of 2595 children with RSV ^14^ infection: mild to moderate infection (*n* = 2435) vs. severe infection (*n* = 160).	Excluded: congenital heart disease, congenital airway malformation, bronchopulmonary dysplasia, severe neurological diseases, immunodeficiency.		NLR ^25^	NLR ^25^ as independent markers for predicting RSV ^14^ severity.
Lian D., et al., 2026 [32]	China	Single-center retrospective cohort	A total of 518 pediatrics hospitalized, aged 28 days to 14 years, primary diagnosed RSV ^14^ infection. Participants were randomly divided into training cohort (*n* = 363) and test cohort (*n* = 155). In the test set, 234 patients did not experience bacterial co-infection, while the remaining 129 did.	Excluded: congenital heart disease, immunodeficiency, other pathogen infections, administered systemic antibiotic for more than 48 h prior.		NLR ^25^, PLR ^27^	Both NLR ^25^ and PLR ^27^ showed significant correlation with bacterial co-infection in RSV ^14^ infection.
Kızılsoy Ö., et al., 2024 [33]	Turkey	Prospective observational	A total of 74 pediatric patients, aged 1 to 24 months, suffering from RSV ^14^ acute bronchiolitis infection: mild (*n* = 57), moderate (*n* = 10), severe (*n* = 7).	Excluded chronic diseases predisposing to severe bronchiolitis: congenital heart disease, cystic fibrosis, bronchopulmonary dysplasia, asthma, recurrent wheezing episodes, immunodeficiency.		NLR ^25^, PLR ^27^, SII ^29^	NLR ^25^ and SII ^29^ had significant value to predict acute bronchiolitis severity due to RSV ^14^.
Yilmaz S., et al., 2022 [34]	Turkey	Retrospective case–control	Children below 18 years old evaluated by RT-PCR ^1^: positive COVID-19 test (*n* = 204) vs. negative COVID-19 test (*n* = 98)	Not restricted	SARS-CoV-2	NLR ^25^, MLR ^26^, ELR ^35^, BLR ^36^, PLR ^27^, MPV/PLT ^37^	ELR ^35^ and BLR ^36^ distinguished pediatric COVID-19 cases from controls, whereas NLR ^25^, MLR ^26^, and PLR ^27^ did not.
Üstündağ Y., et al., 2022 [35]	Turkey	Retrospective case–control	A total of 208 children aged 2 months to 18 years confirmed COVID-19 by RT-PCR ^3^ compared with 117 healthy control.	Excluded: had comorbid either acute or chronic.		NLR ^25^, dNLR ^33^, PLR ^27^, MLR ^26^, EMR ^38^, SII ^29^, SIRI ^30^, AISI ^39^, NLPR ^40^	EMR ^38^ was the best CBC ^1^-derived marker for detecting pediatric COVID-19; NLR ^25^, MLR ^26^, and other inflammatory indices were elevated but showed lower diagnostic performance.
Yazılıtaş F., et al., 2024 [36]	Turkey	Retrospective observational	A total of 486 pediatric patients with RT-PCR ^3^-positive of SARS-CoV-2 infection had routine blood tests and kidney function test.	Excluded: previously suffered from chronic kidney disease and end-stage kidney disease.		NLR ^25^	Elevated NLR ^25^ significantly associated with AKI in COVID-19 infection.
Chelcea R., et al., 2024 [37]	Romania	Longitudinal cohort	This study evaluated a total of 66 pediatric patients in Romania who had recently recovered from acute SARS-CoV-2 infection, dividing them into two cohorts: 28 patients who did not develop secondary pneumonia and 38 patients who did.	Excluded: chronic pulmonary diseases that could affect both lung ultrasound findings and biomarker values.		NLR ^25^, dNLR ^33^	Both NLR ^25^ and dNLR ^33^ significantly predict the development of pneumonia during COVID-19 hospitalization.
Jin Q., et al., 2024 [38]	China	Retrospective cohort	A total of 261 children below 14 years with PCR ^2^-confirmed Omicron SARS-CoV-2 infection underwent a routine blood test.	Not restricted.		NLR ^25^, MLR ^26^, PLR ^27^	Increased NLR ^25^ had the best diagnostic value for severe pediatric COVID-19.
Huang L., et al., 2024 [39]	China	Retrospective cross-sectional comparative	A total of 1420 children hospitalized: SARS-CoV-2 (*n* = 351), influenza A (*n* = 671), RSV ^14^ (*n* = 398), healthy control (*n* = 243). They were grouped by age: <6 months, 6–24 months, 2–6 years, and 6–18 years.	Excluded: chronic systemic diseases (hypertension, respiratory disease, impaired renal function, diabetes, anemia), sepsis, abscess, other pathogen infections, trauma or surgical patients, currently undergoing chemotherapy.	SARS-CoV-2, RSV ^14^, and Influenza A	NLR ^25^, LMR ^34^, MPV/PLT ^37^, LYM*PLT ^41^	LMR ^34^, LYM*PLT ^41^, PLT, and MPV/PLT ^37^ showed the best diagnostic performance, particularly for distinguishing COVID-19 from RSV ^14^ and healthy controls.
Zhu R., et al., 2020 [40]	China	Retrospective case–control	Children with influenza-like symptoms under 18 years old grouped based on RT-PCR ^3^ results: 388 influenza A infection (A+ group), 169 influenza B infection (B+ group), 198 with influenza-like symptoms but no detectable influenza infection (A-/B- group), and 259 healthy children (H group).	Excluded: malignancy, HIV ^4^ infection, severe organ(s) failure, EBV ^47^ infection, bacterial infections, using immunosuppressant prior.	Influenza A, and Influenza B	NLR ^25^, LMR ^34^, MPV/PLT ^37^, LYM*PLT ^41^	NLR ^25^, LMR ^34^, MPV/PLT ^37^, LYM*PLT ^41^ identify influenza infections in cases with influenza-like symptoms and healthy control.
Güneylioğlu M., et al., 2022 [41]	Turkey	Retrospective cohort	A total of 59 patients who examined chest X-ray and thorax ultrasonography were included in this study: empyema (*n* = 16) vs. parapneumonic effusion (*n* = 43).	Excluded: pleural effusion secondary due to malignancy or hydatid cyst rupture, using immunosuppressant and antibiotic within 7 days.	Empyema and parapneumonic effusion	NLR ^25^, PLR ^27^, LMR ^34^, SII ^29^	All markers significantly distinguish parapneumonic effusion and empyema.
Han L., et al., 2026 [42]	China	Retrospective cohort	A total of 388 children aged ≤12 years with culture-confirmed bacterial pneumonia were classified into otitis media group (*n* = 99) and non-otitis media group (*n* = 289) based on development of hospital-acquired otitis media during hospitalization.	Excluded: cardiac impairment, neurosurgical disorders, endocrine diseases, connective tissue diseases, hepatic or renal dysfunction, developmental delay, and malignancy.	Bacterial pneumonia	SII ^29^, AISI ^39^	SII ^29^ and AISI ^39^ independently predict hospital-acquired otitis media in pediatric bacterial pneumonia.
Gauchan E., Adhikari S, 2016 [43]	Nepal	Prospective observational	A total of 654 children aged 1 to 60 months with lower respiratory tract were observed, 285 of them had been confirmed to have bacterial pneumonia.	Not restricted.	Bacterial pneumonia and non-bacterial pneumonia	NLR ^25^	NLR ^25^ had poor value as a CBC ^1^-derived marker for etiology differentiation.
Omran A., et al., 2022 [44]	Egypt	Prospective observational	A total of 52 children aged 2 months–2 years admitted with clinical signs of CAP ^7^: 34 bacterial pneumonia (24 culture-positive + 10 culture-negative diagnosed by neutrophilia + CRP ^22^ + CXR ^20^) vs. 18 viral pneumonia (100% PCR ^2^-positive).	Excluded: complicated pneumonia, major cardiac anomalies, chronic pulmonary diseases, hemodynamically unstable, clinical bronchiolitis, unresolved pneumonia or pneumonia treatment within preceding month.	Bacterial and viral pneumonia	NLR ^25^	The NLR ^25^ itself was sufficiently reliable for distinguishing bacterial pneumonia from viral pneumonia. However, combining the NLR ^25^ and LUS ^21^ improves diagnostic accuracy.
Gambadauro A., et al., 2025 [45]	Italy	Retrospective cohort	A total of 95 infants aged < 12 months hospitalized with viral bronchiolitis during three epidemic seasons.	Excluded: congenital heart disease, hematological disease, chronic neurologic diseases, chronic lung disease, immunodeficiency, trisomy 21, extreme prematurity (<28 weeks), using corticosteroid/antibiotic/antiviral treatment prior.	Viral bronchiolitis	NLR ^25^, PLR ^27^, MLR ^26^, ELR ^35^, LMR ^34^, NPR ^42^, LPR ^43^, LNR ^44^, PNR ^31^, SII ^29^, SIRI ^30^	PLR ^27^, MLR ^26^, and PNR ^31^ were associated with prolonged hospitalization.
Li C., et al., 2025 [46]	China	Retrospective comparative cross-sectional study	A total of 1103 neonates who were diagnosed with CAP ^7^ were divided into two groups based on PNR ^31^ levels (high PNR ^31^ group > 74.49 and low PNR ^31^ group ≤ 74.49).	Excluded: congenital lung abnormalities, congenital heart disease, malignancy, hematological disease.	Community acquired pneumonia	PNR ^31^	PNR ^31^ as a potential predictor for neonatal pneumonia with sepsis.
Wu J., et al., 2021 [47]	China	Retrospective cohort	A total of 5211 children below 18 years old were analyzed comparatively; pneumonia (*n* = 2548) vs. URTI ^23^ (*n* = 2663).	Not restricted.	Community acquired pneumonia vs. upper respiratory tract infection	NLR ^25^, LMR ^34^	NLR ^25^ and LMR ^34^ showed diagnostic usefulness for distinguishing pneumonia from upper respiratory infection in children.
Xu M., et al., 2023 [48]	China	Retrospective cohort	A total of 1032 children, aged 5 to 14 years, divided into: asthma group (*n* = 343), CAP ^7^ group (*n* = 352), asthmatic CAP ^7^ group (*n* = 337).	Excluded: severe CAP ^7^ requiring ICU ^17^, other pathogen infections, solid tumor, cardiovascular disease, acute pulmonary conditions.	Community acquired pneumonia with concomitant asthma exacerbation	NLR ^25^	NLR ^25^ tend to be potential predictor for asthma exacerbation and unimproved CAP ^7^.
Sert S., et al., 2025 [49]	Turkey	Retrospective cross-sectional	A total of 191 hospitalized pediatric patients diagnosed with viral ALRTI ^24^ were examined and analyzed for hematological parameters and systemic inflammatory markers.	Excluded: neonates, children hospitalized within two weeks, congenital heart defects, hematological malignancies, immunodeficiency, bronchopulmonary dysplasia.	Viral pneumonia	NLR ^25^, NMR ^45^, LMR ^34^, PLR ^27^, EMR ^38^, MPVLR ^46^, SII ^29^, SIRI ^30^, AISI ^39^	NLR ^25^, NMR ^45^, PLR ^27^, and MPVLR were potential markers to predict the severity of ALRTI ^24^.

^1^ CBC = complete blood count; ^2^ PCR = polymerase chain reaction; ^3^ RT-PCR = real time polymerase chain reaction; ^4^ HIV = human immunodeficiency virus; ^5^ TB = tuberculosis; ^6^ LRTI = lower respiratory tract infection; ^7^ CAP = community-acquired pneumonia; ^8^ IGRA = interferon-gamma release assay; ^9^ MP = *Mycoplasma pneumoniae*; ^10^ PB = plastic bronchitis; ^11^ MPP = *Mycoplasma pneumoniae* pneumonia; ^12^ SPP = *Streptococcus pneumoniae* pneumonia; ^13^ HMPV = human metapneumovirus; ^14^ RSV = respiratory syncytial virus; ^15^ HAdV = human adenovirus; ^16^ PICU = pediatric intensive care unit; ^17^ ICU = intensive care unit; ^18^ DNA = deoxyribonucleic acid; ^19^ RNA = ribonucleic acid; ^20^ CXR = chest X-ray; ^21^ LUS = lung ultrasound; ^22^ CRP = C-reactive protein; ^23^ URTI = upper respiratory tract infection; ^24^ ALRTI = acute lower respiratory tract infections; ^25^ NLR = neutrophil-to-lymphocyte ratio; ^26^ MLR = monocyte-to-lymphocyte ratio; ^27^ PLR = platelet-to-lymphocyte ratio; ^28^ NMLR = neutrophil-monocyte-to-lymphocyte ratio; ^29^ SII = systemic immune-inflammation index; ^30^ SIRI = systemic inflammatory response index; ^31^ PNR = platelet-to-neutrophil ratio; ^32^ NEUT% = neutrophil percentage; ^33^ dNLR = derived neutrophil-to-lymphocyte ratio; ^34^ LMR = lymphocyte-to-monocyte ratio; ^35^ ELR = eosinophil-to-lymphocyte ratio; ^36^ BLR = basophil-to-lymphocyte ratio; ^37^ MPV/PLT = mean platelet volume-to-platelet count; ^38^ EMR = eosinophil-to-monocyte ratio; ^39^ AISI = aggregate index of systemic inflammation; ^40^ NLPR = neutrophil-to-lymphocyte-platelet ratio; ^41^ LYM*PLT = lymphocyte multiplied by platelet; ^42^ NPR = neutrophil-to-platelet ratio; ^43^ LPR = lymphocyte-to-platelet ratio; ^44^ LNR = lymphocyte-to-neutrophil ratio (LNR = lymphocyte count/neutrophil count); ^45^ NMR = neutrophil-to-monocyte ratio; ^46^ MPVLR = mean platelet volume-to-lymphocyte ratio; ^47^ EBV = Epstein–Barr virus.

**Table 3:** Reported performance values of CBC-derived markers in pediatric bacterial LRTI.

Etiology	Study	Marker	Target (Comparison)	Cut-Off	AUC	Sensitivity	Specificity	OR/HR (95% CI)
*Bordetella pertussis*	Li C., et al., 2026 [4]	NLR ^1^	Severity assessment (severe pertussis)	0.475	0.745	66.70%	75.50%	OR ^2^ 1.884 (1.157–3.066)
	Tang J., et al., 2025 [5]	NLR ^1^	Diagnosis (6 months; pertussis vs. non-pertussis)	0.43	0.903	85.40%	84.40%	*p* < 0.001
		MLR ^3^		0.12	0.773	58.40%	87.50%	*p* < 0.001
		PLR ^4^		-	0.555	-	-	*p* < 0.001
		NMLR ^5^		0.49	0.904	89.90%	82.80%	*p* < 0.001
		SII ^6^		180.64	0.816	74.20%	78.10%	*p* < 0.001
		SIRI ^7^		0.25	0.865	79.80%	87.50%	*p* < 0.001
		NLR ^1^	Diagnosis (6 months-1 year; pertussis vs. non-pertussis)	0.46	0.88	80.90%	86.00%	*p* < 0.001
		MLR ^3^		0.10	0.780	68.10%	80.20%	*p* < 0.001
		PLR ^4^		-	0.618	-	-	*p* < 0.001
		NMLR ^5^		0.54	0.886	83.00%	84.90%	*p* < 0.001
		SII ^6^		161.28	0.860	78.70%	82.60%	*p* < 0.001
		SIRI ^7^		0.29	0.892	78.70%	89.50%	*p* < 0.001
		NLR ^1^	Diagnosis (1–2 years; pertussis vs. non-pertussis)	0.70	0.852	79.50%	79.90%	*p* < 0.001
		MLR ^3^		-	0.694	-	-	*p* < 0.001
		PLR ^4^		-	0.677	-	-	*p* < 0.001
		NMLR ^5^		0.81	0.843	81.80%	78.90%	*p* < 0.001
		SII ^6^		246.25	0.838	70.50%	84.40%	*p* < 0.001
		SIRI ^7^		0.37	0.801	75.00%	75.90%	*p* < 0.001
		NLR ^1^	Diagnosis (2–6 years; pertussis vs. non-pertussis)	-	0.600	-	-	*p* < 0.001
		MLR ^3^		-	0.570	-	-	*p* < 0.001
		PLR ^4^		-	0.434	-	-	*p* < 0.001
		NMLR ^5^		-	0.599	-	-	*p* < 0.001
		SII ^6^		-	0.594	-	-	*p* < 0.001
		SIRI ^7^		-	0.657	-	-	*p* < 0.001
		NLR ^1^	Diagnosis (6–13 years; pertussis vs. non-pertussis)	-	0.602	-	-	*p* < 0.001
		MLR ^3^		-	0.573	-	-	*p* < 0.001
		PLR ^4^		-	0.766	-	-	*p* < 0.001
		NMLR ^5^		-	0.601	-	-	*p* < 0.001
		SII ^6^		-	0.568	-	-	*p* < 0.001
		SIRI ^7^		-	0.449	-	-	*p* < 0.001
		NLR ^1^	Diagnosis (13–18 years; pertussis vs. non-pertussis)	-	0.529	-	-	*p* < 0.001
		MLR ^3^		-	0.688	-	-	*p* < 0.001
		PLR ^4^		90.92	0.743	88.20%	75.00%	*p* < 0.001
		NMLR ^5^		-	0.551	-	-	*p* < 0.001
		SII ^6^		-	0.496	-	-	*p* < 0.001
		SIRI ^7^		-	0.467	-	-	*p* < 0.001
	Zhou J., et al., 2025 [6]	NLR ^1^	Diagnosis (pertussis vs. non-pertussis)	-	-	-	-	*p* = 0.005
		MLR ^3^		-	-	-	-	*p* < 0.001
		PLR ^4^		-	-	-	-	*p* < 0.001
		SII ^6^		-	-	-	-	*p* = 0.047
		SIRI ^7^		-	-	-	-	*p* = 0.060
*Mycobacterium* *tuberculosis*	Choudhary R., et al., 2019 [7]	MLR ^3^	Diagnosis (TB ^8^ vs. non-TB ^8^ among HIV ^15^-infected children)	0.378	-	77.00%	78.00%	*p* < 0.01
	Kissling M., et al., 2023 [8]	NLR ^1^	Diagnosis (TB ^8^ vs. non-TB ^8^)	Median 2.0 (TB ^8^) vs. 0.3 (non-TB ^8^)	-	-	-	*p* < 0.001
		MLR ^3^		Median 1.4 (TB ^8^) vs. 0.2 (non-TB ^8^)	-	-	-	*p* < 0.001
		NMLR ^5^		-	-	-	-	Significant (full data in primary source)
	Ma W., et al., 2024 [9]	NLR ^1^	Severity assessment (severe TB ^8^)	-	-	-	-	1.354 (1.024–1.789)
	Rees C., et al., 2020 [10]	NLR ^1^	Diagnosis (TB ^8^ vs. non-TB ^8^)	93	0.56	45.00%	81.00%	*p* = 0.08
		MLR ^3^		0.18	0.56	68.00%	51.00%	*p* = 0.10
		PLR ^4^		0.53	0.59	36.00%	95.00%	*p* = 0.28
*Mycoplasma* *pneumoniae*	Feng M., et al., 2026 [11]	NLR ^1^	Severity assessment (MPP ^9^-associated plastic bronchitis)	-	-	-	-	Significant higher in PB ^16^ group (*p* < 0.001)
		MLR ^3^		-	-	-	-	*p* = 0.057
		PLR ^4^		143.621	0.759	65.91%	76.14%	1.011 (1.002–1.020)
		SII ^6^		896.206	0.77	70.45%	76.14%	0.999 (0.998–1.000)
		SIRI ^7^		-	-	-	-	*p* = 0.068
	Guo X., et al., 2025 [12]	NLR ^1^	Severity assessment (severe MPP ^9^)	-	-	-	-	0.838 (0.433–1.623)
		MLR ^3^		-	-	-	-	0.734 (0–1240.5)
		PLR ^4^		-	-	-	-	1.001 (0.985–1.017)
		SII ^6^		689.85	0.883	69.90%	88.10%	1.006 (1.001–1.010)
		SIRI ^7^		-	-	-	-	1.221 (0.313–4.762)
	Li D., et al., 2023 [13]	NLR ^1^ (NP ^17^)	Severity and complication prediction: necrotizing pneumonia and refractory MPP ^9^	-	0.888	-	-	1.153 (1.022–1.300)
		NLR ^1^ (RMPP ^10^)		-	0.736	-	-	1.246 (1.102–1.408)
	Shao L., et al., 2025 [14]	NLR ^1^	Diagnosis (MPP ^9^ vs. non-MPP ^9^)	1.61	0.867	75.00%	86.00%	8.424 (5.479–12.954)
		MLR ^3^		0.20	0.876	80.20%	84.70%	6.061 (4.259–8.624)
		PLR ^4^		129.51	0.697	54.00%	79.30%	1.017 (1.012–1.023)
		SII ^6^		485.38	0.815	66.50%	84.00%	1.005 (1.004–1.007)
		SIRI ^7^		0.84	0.892	75.70%	92.00%	1.467 (1.355–1.589)
	Wang S., et al., 2024 [15]	NLR ^1^	Severity assessment (severe MPP ^9^)	2.69	0.813	74.40%	77.40%	*p* < 0.001
		PLR ^4^		139.19	0.804	70.50%	78.30%	*p* < 0.001
		SII ^6^		699	0.94	87.60%	98.70%	*p* < 0.001
		SIRI ^7^		1.15	0.839	74.40%	82.30%	*p* < 0.001
	Zhang K., et al., 2026 [16]	NLR ^1^	Diagnosis (MP ^11^ vs. non-MP ^11^)	1.36	0.624	79.80%	55.80%	0.839 (0.784–0.894)
*Mycoplasma* *pneumoniae* vs. Influenza	Chen J., et al., 2024 [17]	PLR ^4^	Etiology differentiation (MP ^11^ vs. Influenza)	-	0.708	-	-	*p* < 0.001
*Mycoplasma* *pneumoniae* vs. RSV ^12^	Zhou H., Qian K., 2025 [18]	SII ^6^	Etiology differentiation (MP ^11^ vs. RSV ^12^)	-	-	-	-	Significantly higher in MP ^11^ group: <6 months *p* = 0.012; 6 months-1 year *p* = 0.108; >1 year *p* = 0.015
		SIRI ^7^		-	-	-	-	Significantly higher in MP ^11^ group: <6 months *p* = 0.034; 6 months-1 year *p* = 0.249; >1 year *p* = 0.014
*Mycoplasma* *pneumoniae* vs. bacterial pneumonia	Zheng H., et al., 2022 [19]	NLR ^1^	Diagnosis (bacterial pneumonia vs. healthy control)	-	0.767 (0.664–0.850)	-	-	*p* < 0.05
		PLR ^4^		71.22	0.898 (0.815–0.953)	88.36%	84.09%	*p* < 0.05
		NLR ^1^	Diagnosis (bacterial pneumonia vs. MP ^11^)	-	0.685 (0.577–0.780)	-	-	*p* < 0.05
		PLR ^4^		71.26	0.803	70.45%	84.63%	*p* < 0.05
*Mycoplasma* *pneumoniae* vs. viral pneumonia	Wan Y., et al., 2026 [20]	NLR ^1^	Etiology differentiation (MP ^11^ vs. VP ^14^)	1.44	0.77	73.00%	72.00%	1.67 (1.08–2.58)
		PLR ^4^		-	-	-	-	*p* = 0.36
*Streptococcus* *pneumoniae*	Bao M., et al., 2026 [21]	NLR ^1^		1.395	0.676	70.90%	57.40%	*p* < 0.001
		PLR ^4^		-	-	-	-	*p* = 0.001
		PNR ^13^		-	-	-	-	*p* = 0.018

^1^ NLR = neutrophil-to-lymphocyte ratio; ^2^ OR = odds ratio; ^3^ MLR = monocyte-to-lymphocyte ratio; ^4^ PLR = platelet-to-lymphocyte ratio; ^5^ NMLR = neutrophil-monocyte-to-lymphocyte ratio; ^6^ SII = systemic immune-inflammation index; ^7^ SIRI = systemic inflammatory response index; ^8^ TB = tuberculosis; ^9^ MPP = *Mycoplasma pneumoniae* pneumonia; ^10^ RMPP = refractory *Mycoplasma pneumoniae* pneumonia; ^11^ MP = mycoplasma pneumoniae; ^12^ RSV = respiratory syncytial virus; ^13^ PNR = platelet-to-neutrophil ratio; ^14^ VP = viral pneumonia; ^15^ HIV = human immunodeficiency virus; ^16^ PB = plastic bronchitis; ^17^ NP = necrotizing pneumonia. Where a primary study reported only a significance test for a marker, the *p*-value is given and the cut-off, AUC, sensitivity, and specificity columns are marked as not reported (-); such rows indicate a difference between groups rather than discriminative performance. Confidence intervals are presented, where available, with the estimate to which they refer in the primary study.

**Table 4:** Reported performance values of CBC-derived markers in pediatric viral LRTI.

Etiology	Study	Marker	Target (Comparison)	Cut-Off	AUC	Sensitivity	Specificity	OR/HR (95% CI)
Human adenovirus	Wang N., et al., 2024 [22]	NEUT% ^1^	Severity assessment (severe adenovirus pneumonia)	47.60%	0.728	70.10%	70.40%	OR^2^ = 1.034
	Xu H., et al., 2025 [23]	NLR ^3^	Severity assessment (severe adenovirus pneumonia)	-	-	-	-	*p* = 0.2481
		PLR ^4^		-	0.599	-	-	*p* < 0.05
		PNR ^5^		-	-	-	-	*p* = 0.5942
Human metapneumovirus	Guo Y., et al., 2025 [24]	NLR ^3^	Severity assessment (severe HMPV ^6^ pneumonia)	-	-	-	-	*p* = 0.03
		PLR ^4^		-	-	-	-	*p* = 0.001
		PNR ^5^		-	-	-	-	*p* > 0.05
	Liu L., et al., 2026 [25]	NLR ^3^	Severity assessment (severe HMPV ^6^ pneumonia)	-	-	-	-	1.75 (1.26–2.43)
Human parainfluenza viruses	Chu F., et al., 2023 [26]	NLR ^3^	Outcome prediction (PICU ^7^ admissions)	-	-	-	-	*p* < 0.001
		dNLR ^8^		-	-	-	-	*p* < 0.001
		PLR ^4^		-	-	-	-	1.009 (1.002–1.017)
		LMR ^9^		-	-	-	-	*p* < 0.001
RSV ^10^	Okay Z., et al., 2024 [27]	NLR ^3^	Severity assessment (severe RSV ^10^ pneumonia)	1.49	-	62.20%	62.20%	*p* < 0.001
	Okuyan O., et al., 2023 [28]	NLR ^3^	Diagnosis (RSV ^10^ vs. non-RSV ^10^)	0.85	0.706 (0.636–0.776)	51.30%	95.50%	-
		PLR ^4^		73	0.779 (0.722–0.836)	50.40%	90.50%	-
		SII ^11^		280	0.705 (0.633–0.776)	56.60%	93.90%	-
	Pan J., et al., 2026 [29]	NLR ^3^	Severity assessment (severe RSV ^10^ pneumonia)	-	-	-	-	*p* < 0.001
	Burrack N., et al., 2023 [30]	NLR ^3^	Outcome prediction (PICU ^7^ admissions)	0.67	0.72 (0.679–0.784)	71.00%	35.00%	-
		MLR ^12^		0.34	0.73 (0.681–0.789)	54.00%	72.00%	-
	Li W., et al., 2026 [31]	NLR ^3^	Severity assessment (severe RSV ^10^ pneumonia)	-	0.847	-	-	1.126 (1.087–1.360)
	Lian D., et al., 2026 [32]	NLR ^3^	Severity assessment (bacterial co-infection)	-	0.811	-	-	2.13 (1.64–2.79)
		PLR ^4^		-	-	-	-	*p* < 0.001
	Kızılsoy Ö., et al., 2024 [33]	NLR ^3^	Severity assessment (severe RSV ^10^ bronchiolitis)	1.06	0.80	85.70%	40.30%	0.649–0.950
		PLR ^4^		-	-	-	-	*p* = 0.204
		SII ^11^		409.01	0.83	85.70%	40.30%	0.607–0.999
SARS-CoV-2 ^21^	Yilmaz S., et al., 2022 [34]	NLR ^3^	Diagnosis (COVID-19 ^22^ vs. non-COVID-19 ^22^)	2.0	0.568	71.40%	35.80%	*p* = 0.133
		MLR ^12^		0.321	0.501	66.30%	31.10%	*p* = 0.805
		ELR ^13^		0.058	0.613	78.60%	39.60%	*p* = 0.005
		BLR ^14^		0.01	0.693	88.80%	34.00%	*p* < 0.001
		PLR ^4^		763.159	0.500	59.20%	37.70%	*p* = 0.931
		MPV/PLT ^15^		3.385	0.675	53.10%	31.10%	*p* = 0.159
	Üstündağ Y., et al., 2022 [35]	NLR ^3^	Diagnosis (COVID-19 ^22^ vs. non-COVID-19 ^22^)	-	0.612	-	-	*p* = 0.003
		dNLR ^8^		-	0.603	-	-	*p* = 0.05
		PLR ^4^		-	0.574	-	-	*p* = 0.046
		MLR ^12^		-	0.719	-	-	*p* < 0.01
		EMR ^16^		0.26	0.777	77.40%	71.10%	*p* < 0.01
		SII ^11^		-	0.578	-	-	*p* = 0.034
		SIRI ^17^		-	0.672	-	-	*p* < 0.01
		AISI ^18^			0.641			*p* < 0.01
		NLPR ^19^		-	0.635	-	-	*p* < 0.01
	Yazılıtaş F., et al., 2024 [36]	NLR ^3^	Severity assessment (COVID-19 ^22^ -associated AKI ^23^)	-	-	-	-	*p* = 0.003
	Chelcea R., et al., 2024 [37]	NLR ^3^	Severity assessment (severe COVID-19 ^22^ pneumonia)	3.84	0.485	52.70%	57.10%	*p* = 0.327
		dNLR ^8^		1.88	0.802	77.00%	85.70%	*p* < 0.001
	Jin Q., et al., 2024 [38]	NLR ^3^	Severity assessment (severe COVID-19 ^22^ pneumonia)	-	0.643	-	-	1.130 (1.021–1.251)
		MLR ^12^		-	-	-	-	*p* = 0.193
		PLR ^4^		-	-	-	-	*p* = 0.352
SARS-CoV-2 ^21^, RSV ^10^, and Influenza A	Huang L., et al., 2024 [39]	LMR ^9^	Diagnosis (SARS-CoV-2 ^21^ vs. healthy control) aged below 6 months	8.58	0.860 (0.797–0.909)	69.50%	100%	-
		LYM*PLT ^20^		1742.3	0.860 (0.796–0.909)	70.80%	100%	-
		MPV/PLT ^15^		0.0235	0.911 (0.856–0.950)	77.30%	100%	-
		NLR ^3^	Diagnosis (SARS-CoV-2 ^21^ vs. Influenza A) aged below 6 months	0.312	0.662 (0.587–0.731)	55.20%	77.30%	-
		LMR ^9^		4.52	0.678 (0.604–0.746)	44.80%	86.40%	-
		LYM*PLT ^20^	Diagnosis (SARS-CoV-2 ^21^ vs. RSV ^10^) aged below 6 months	1642.2	0.767 (0.703–0.823)	66.90%	74.50%	-
		MPV/PLT ^15^		0.0306	0.760 (0.696–0.817)	51.90%	88.20%	-
		LMR ^9^	Diagnosis (SARS-CoV-2 ^21^ vs. healthy control) aged 6 to 24 months	6.09	0.695 (0.622–0.762)	43.20%	97.60%	-
		LYM*PLT ^20^		1246.3	0.744 (0.673–0.806)	61.10%	85.50%	-
		MPV/PLT ^15^		0.0325	0.752 (0.681–0.813)	61.10%	79.50%	-
		NLR ^3^	Diagnosis (SARS-CoV-2 ^21^ vs. Influenza A) aged 6 to 24 months	0.3245	0.564 (0.493–0.633)	66.30%	50.90%	-
		LYM*PLT ^20^	Diagnosis (SARS-CoV-2 ^21^ vs. RSV ^10^) aged 6 to 24 months	702	0.715 (0.652–0.772)	40.00%	93.60%	-
		MPV/PLT ^15^		0.031	0.720 (0.658–0.777)	66.30%	68.60%	-
		LMR ^9^	Diagnosis (SARS-CoV-2 ^21^ vs. healthy control) aged 2 to 6 years	4.9138	0.842 (0.749–0.911)	77.80%	82.70%	-
		LYM*PLT ^20^		601.6	0.810 (0.712–0.886)	69.40%	86.50%	-
		MPV/PLT ^15^		0.0406	0.733 (0.628–0.821)	50.00%	84.60%	-
		LMR ^9^	Diagnosis (SARS-CoV-2 ^21^ vs. Influenza A) aged 2 to 6 years	4.9138	0.610 (0.550–0.668)	77.80%	50.80%	-
		MPV/PLT ^15^		0.0428	0.662 (0.550–0.668)	69.40%	68.20%	-
		LYM*PLT ^20^	Diagnosis (SARS-CoV-2 ^21^ vs. RSV ^10^) aged 2 to 6 years	490.68	0.839 (0.691–0.935)	63.90%	100%	-
		LMR ^9^	Diagnosis (SARS-CoV-2 ^21^ vs. healthy control) aged 6 to 18 years	7.3286	0.813 (0.746–0.870)	84.80%	73.00%	-
		LYM*PLT ^20^		630.72	0.844 (0.779–0.895)	66.70%	90.00%	-
		MPV/PLT ^15^	Diagnosis (SARS-CoV-2 ^21^ vs. Influenza A) aged 6 to 18 years	0.0429	0.613 (0.561–0.663)	60.60%	63.30%	-
Influenza A and Influenza B	Zhu R., et al., 2020 [40]	NLR ^3^	Diagnosis (Influenza A, children aged 1 to 6 years old)	-	-	-	-	*p* < 0.01
		LMR ^9^		3.75	0.886	81.87%	84.31%	*p* < 0.01
		MPV/PLT ^15^		-	-	-	-	*p* < 0.01
		LYM*PLT ^20^		680.48	0.958	90.67%	89.66%	*p* < 0.01
		NLR ^3^	Diagnosis (Influenza A, children aged above 6 years old)	-	-	-	-	*p* < 0.01
		LMR ^9^		3.09	0.975	90.40%	95.24%	*p* < 0.01
		MPV/PLT ^15^		-	-	-	-	*p* < 0.01
		LYM*PLT ^20^		-	-	-	-	*p* < 0.01
		NLR ^3^	Diagnosis (Influenza B, children aged 1 to 6 years old)	-	-	-	-	*p* < 0.01
		LMR ^9^		4.47	0.918	86.79%	89.66%	*p* < 0.01
		MPV/PLT ^15^		-	-	-	-	*p* < 0.01
		LYM*PLT ^20^		-	-	-	-	*p* < 0.01
		NLR ^3^	Diagnosis (Influenza B, children aged above 6 years old)	-	-	-	-	*p* < 0.01
		LMR ^9^		3.48	0.954	90.27%	92.38%	*p* < 0.01
		MPV/PLT ^15^		-	-	-	-	*p* < 0.01
		LYM*PLT ^20^		-	-	-	-	*p* < 0.01

^1^ NEUT% = neutrophil percentage; ^2^ OR = odds ratio; ^3^ NLR = neutrophil-to-lymphocyte ratio; ^4^ PLR = platelet-to-lymphocyte ratio; ^5^ PNR = platelet-to-neutrophil ratio; ^6^ HMPV = human metapneumovirus; ^7^ PICU = pediatric intensive care unit; ^8^ dNLR = derived neutrophil-to-lymphocyte ratio; ^9^ LMR = lymphocyte-to-monocyte ratio (LMR = lymphocyte count/monocyte count); ^10^ RSV = respiratory syncytial virus; ^11^ SII = systemic immune-inflammation index; ^12^ MLR = monocyte-to-lymphocyte ratio; ^13^ ELR = eosinophil-to-lymphocyte ratio; ^14^ BLR = basophil-to-lymphocyte ratio; ^15^ MPV/PLT = mean platelet volume-to-platelet count; ^16^ EMR = eosinophil-to-monocyte ratio; ^17^ SIRI = systemic inflammatory response index; ^18^ AISI = aggregate index of systemic inflammation; ^19^ NLPR = neutrophil-to-lymphocyte-platelet ratio; ^20^ LYM*PLT = lymphocyte multiplied by platelet; ^21^ SARS-CoV-2 = severe acute respiratory syndrome coronavirus 2; ^22^ COVID-19 = coronavirus disease 2019; ^23^ AKI = acute kidney injury. Where a primary study reported only a significance test for a marker, the *p*-value is given and the cut-off, AUC, sensitivity, and specificity columns are marked as not reported (-); such rows indicate a difference between groups rather than discriminative performance. Confidence intervals are presented, where available, with the estimate to which they refer in the primary study.

**Table 5:** Reported performance values of CBC-derived markers in pediatric mixed-pathogen LRTI.

Etiology	Study	Marker	Target (Comparison)	Cut-off	AUC ^14^	Sensitivity	Specificity	OR/HR (95% CI)
Mixed pathogen infection, dominated by *Streptococcus pneumoniae, Streptococcus pyogenes*, and *Staphylococcus aureus*	Güneylioğlu M., et al., 2022 [41]	NLR ^1^	Diagnosis (empyema vs. parapneumonic effusion)	7.63	0.83 (0.71–0.94)	68.80%	66.70%	4.55
		PLR ^3^		206.77	0.79	68.80%	66.70%	4.55
		LMR ^4^		2.26	0.82 (0.69–0.94)	75.00%	69.80%	6.92
		SII ^5^		2609	0.84 (0.72–0.96)	75.00%	71.70%	8.72
Mixed bacterial infection: *Haemophilus influenzae, Streptococcus pneumoniae, Staphylococcus aureus*, and *Moraxella catarrhalis*	Han L., et al., 2026 [42]	SII ^5^	Severity assessment (hospital-acquired otitis media among bacterial pneumonia; AUC of a multivariable nomogram containing the log2-transformed marker)	-	0.827 (0.780–0.875)	76.80%	73.70%	1.38 (1.05–1.82)
		AISI ^6^		-	0.834 (0.788–0.881)	75.80%	74.70%	1.37 (1.07–1.78)
Bacterial pneumonia, and other pathogens related with pneumonia	Gauchan E., Adhikari S, 2016 [43]	NLR ^1^	Etiology differentiation (bacterial pneumonia vs. other pathogen infection)	1.28	0.56 (0.52–0.60)	45.60%	64.00%	*p* = 0.009
Bacterial and viral pneumonia	Omran A., et al., 2022 [44]	NLR ^1^	Etiology differentiation (bacterial vs. viral pneumonia)	-	-	73.50%	94.40%	*p* = 0.0001
Viral bronchiolitis, predominantly RSV, Rhinovirus, Metapneumovirus, Adenovirus, SARS-CoV-2, Influenza, Parainfluenza, Enterovirus, and Coronavirus OC43	Gambadauro A., et al., 2025 [45]	NLR ^1^	Outcome prediction (bronchiolitis risk of admission score)	-	-	-	-	*p* = 0.48
		PLR ^3^		-	-	-	-	*p* = 0.66
		MLR ^2^		-	-	-	-	*p* = 0.18
		ELR ^7^		-	-	-	-	*p* = 0.66
		LMR ^4^		-	-	-	-	*p* = 0.32
		NPR ^8^		-	-	-	-	*p* = 0.89
		LPR ^9^		-	-	-	-	*p* = 0.39
		LNR ^10^		-	-	-	-	*p* = 0.17
		PNR ^11^		-	-	-	-	*p* = 0.17
		SII ^5^		-	-	-	-	*p* = 0.68
		SIRI ^12^		-	-	-	-	*p* = 0.76
Mixed pathogen infection	Li C., et al., 2025 [46]	PNR ^11^	Severity assessment (pneumonia with sepsis)	60.91	0.76 (0.73–0.80)	68.18%	74.40%	0.995 (0.992–0.998)
Mixed pathogen infection	Wu J., et al., 2021 [47]	NLR ^1^	Diagnosis (pneumonia vs. upper respiratory tract infection)	-	0.71 (0.68–0.74)	-	-	-
		LMR ^4^		-	0.76 (0.73–0.79)	-	-	-
Mixed pathogen infection	Xu M., et al., 2023 [48]	NLR ^1^	Diagnosis (community-acquired pneumonia in asthma exacerbation)	4.15	0.711 (0.670–0.751)	56.90%	90.10%	2.5 (1.4–3.6)
Viral pneumonia, predominantly RSV, influenza A, influenza B, adenovirus, parainfluenza, human rhinovirus, enterovirus, human metapneumovirus, and human bocavirus	Sert S., et al., 2025 [49]	NLR ^1^	Severity assessment (severe viral acute lower respiratory tract infections)	-	-	-	-	*p* = 0.001
		NMR		-	-	-	-	*p* = 0.001
		LMR ^4^		-	-	-	-	*p* = 0.349
		PLR ^3^		-	-	-	-	*p* = 0.001
		EMR		-	-	-	-	*p* = 0.619
		MPVLR ^13^		-	-	-	-	*p* < 0.0001
		SII ^5^		-	-	-	-	*p* = 0.117
		SIRI ^12^		-	-	-	-	*p* = 0.678
		AISI ^6^		-	-	-	-	*p* = 0.258

^1^ NLR = neutrophil-to-lymphocyte ratio; ^2^ MLR = monocyte-to-lymphocyte ratio; ^3^ PLR = platelet-to-lymphocyte ratio; ^4^ LMR = lymphocyte-to-monocyte ratio; ^5^ SII = systemic immune-inflammation index; ^6^ AISI = aggregate index of systemic inflammation; ^7^ ELR = eosinophil-to-lymphocyte ratio; ^8^ NPR = neutrophil-to-platelet ratio; ^9^ LPR = lymphocyte-to-platelet ratio; ^10^ LNR = lymphocyte-to-neutrophil ratio; ^11^ PNR = platelet-to-neutrophil ratio; ^12^ SIRI = systemic inflammatory response index; ^13^ MPVLR = mean platelet volume-to-lymphocyte ratio; ^14^ AUC = area under curve.

## Data Availability

No new data were created or analyzed in this study. Data sharing is not applicable to this article.

## Supplementary Materials

The following supporting information can be downloaded at https://www.mdpi.com/article/10.3390/pathogens15090956/s1. Table S1: Diagnostic value of CBC-derived inflammatory markers across pediatric LRTI pathogens; Table S2: Etiological differentiation using CBC-derived inflammatory markers across pediatric LRTI pathogens; Table S3: The effectiveness of CBC-derived inflammatory markers in severity assessment of pediatric LRTI pathogens; Table S4: The effectiveness of CBC-derived inflammatory markers in predicting the outcome of pediatric LRTI pathogens. Table S5: CBC-derived inflammatory markers in pediatric bacterial LRTI, organized by cell wall structure. Quantitative performance estimates for each marker are provided in Table 3 of the main text; Table S6: CBC-derived inflammatory markers in pediatric viral LRTI, organized by genome type and envelope. Quantitative performance estimates for each marker are provided in Table 4 of the main text; Table S7: CBC-derived inflammatory markers in mixed-pathogen pediatric LRTI. Quantitative performance estimates for each marker are provided in Table 5 of the main text; Table S8: The Boolean codes for PubMed advanced search. The final PubMed search was conducted on 9 August 2026.

## Abbreviations

The following abbreviations are used in this manuscript; the calculation formulas for the CBC-derived ratios are given in Table 1:

ACE2	Angiotensin-converting enzyme 2
ADP	Adenosine diphosphate
AISI	Aggregate index of systemic inflammation
AKI	Acute kidney injury
ALRTI	Acute lower respiratory tract infection
ARDS	Acute respiratory distress syndrome
AUC	Area under the curve
BLR	Basophil-to-lymphocyte ratio
BP	*Bordetella pertussis*
cAMP	Cyclic adenosine monophosphate
CAP	Community-acquired pneumonia
CARDS	Community-acquired respiratory distress syndrome
CBC	Complete blood count
CCL2	C-C motif chemokine ligand 2
CD4	Cluster of differentiation 4
CD8	Cluster of differentiation 8
CI	Confidence interval
COVID-19	Coronavirus disease 2019
CRP	C-reactive protein
CXCL10	C-X-C motif chemokine ligand 10
CXCL8	C-X-C motif chemokine ligand 8
CXR	Chest X-ray
DNA	Deoxyribonucleic acid
dNLR	Derived neutrophil-to-lymphocyte ratio
EBV	Epstein–Barr virus
ELR	Eosinophil-to-lymphocyte ratio
EMR	Eosinophil-to-monocyte ratio
H1N1	Influenza A virus subtype H1N1
H3N2	Influenza A virus subtype H3N2
HAdV	Human adenovirus
HAP	Hospital-acquired pneumonia
HC	Healthy control
HIV	Human immunodeficiency virus
HMPV	Human metapneumovirus
HPIV	Human parainfluenza virus
HR	Hazard ratio
ICU	Intensive care unit
IFN-α	Interferon alpha
IFN-β	Interferon beta
IFN-γ	Interferon gamma
IGRA	Interferon-gamma release assay
IL-1β	Interleukin-1 beta
IL-33	Interleukin-33
IL-6	Interleukin-6
IL-8	Interleukin-8
InfA	Influenza A
InfB	Influenza B
IQR	Interquartile range
IRF3	Interferon regulatory factor 3
IRF7	Interferon regulatory factor 7
LMR	Lymphocyte-to-monocyte ratio
LNR	Lymphocyte-to-neutrophil ratio
LOS	Length of stay
LPR	Lymphocyte-to-platelet ratio
LRTI	Lower respiratory tract infection
LUS	Lung ultrasound
LYM*PLT	Lymphocyte multiplied by platelet
MBI	Mixed bacterial infection
MLR	Monocyte-to-lymphocyte ratio
MP	*Mycoplasma pneumoniae*
MPI	Mixed pathogen infection
MPP	*Mycoplasma pneumoniae* pneumonia
MPVLR	Mean platelet volume-to-lymphocyte ratio
MPV/PLT	Mean platelet volume-to-platelet count
MTB	*Mycobacterium tuberculosis*
MVI	Mixed viral infection
MyD88	Myeloid differentiation primary response 88
NEUT%	Neutrophil percentage
NF-κB	Nuclear factor kappa B
NK	Natural killer
NLPR	Neutrophil-to-lymphocyte-platelet ratio
NLR	Neutrophil-to-lymphocyte ratio
NMLR	Neutrophil-monocyte-to-lymphocyte ratio
NMR	Neutrophil-to-monocyte ratio
NP	Necrotizing pneumonia
NPR	Neutrophil-to-platelet ratio
NPV	Negative predictive value
OM	Otitis media
OR	Odds ratio
PB	Plastic bronchitis
PCR	Polymerase chain reaction
PCT	Procalcitonin
PICU	Pediatric intensive care unit
PLR	Platelet-to-lymphocyte ratio
PNR	Platelet-to-neutrophil ratio
PPV	Positive predictive value
PRESS	Pediatric respiratory severity score
RBC	Red blood cell
RMPP	Refractory *Mycoplasma pneumoniae* pneumonia
RNA	Ribonucleic acid
RR	Relative risk
RSV	Respiratory syncytial virus
RT-PCR	Reverse transcription polymerase chain reaction
SAA	Serum amyloid A
SARS-CoV-2	Severe acute respiratory syndrome coronavirus 2
SC2	SARS-CoV-2
SII	Systemic immune-inflammation index
SIRI	Systemic inflammatory response index
SMPP	Severe *Mycoplasma pneumoniae* pneumonia
SP	*Streptococcus pneumoniae*
SPP	*Streptococcus pneumoniae* pneumonia
TB	Tuberculosis
Th2	T helper type 2
TLR2	Toll-like receptor 2
TMPRSS2	Transmembrane serine protease 2
TNF-α	Tumor necrosis factor alpha
URTI	Upper respiratory tract infection
VP	Viral pneumonia
WBC	White blood cell
YKL-40	Chitinase-3-like protein 1
