# Supplementary material for: Complete Blood Count-Derived Inflammatory Markers in Pediatric Lower Respiratory Tract Infection: A Narrative Review
Source: Pathogens. 2026 Sep 8;15(9):956. doi: 10.3390/pathogens15090956 (PMC13610868; doi:10.3390/pathogens15090956)
Supplement: Supplementary file 1 [file pathogens-15-00956-s001.zip › pathogens-4508309-supplementary.pdf]

Supplementary Materials

Table S1. Diagnostic value of CBC-derived inflammatory markers across pediatric LRTI pathogens.

Pathogen	NLR[5]	MLR[6]	PLR[7]	NMLR[8]	SII[9]	SIRI[10]	LMR[11]	MPV/PLT[12]	LYM*PLT[13]	dNLR[14]	AISI[15]	NLPR[16]	EMR[17]	ELR[18]	BLR[19]
Bordetella pertussis (Tan J. et al., 2025; Zhou J., et al., 2025)	↕	↕	↓	↑	↕	×									
Mycobacterium tuberculosis (Kissling M., et al., 2023; Choudhary R., et al., 2019; Rees et C., et al., 2020)	↕	×	×	↑											
Mycoplasma pneumonia (Zhang K., et al., 2026; Shao l., et al., 2025; Feng M., et al., 2026; Zheng H., et al., 2022)	↕	↕	↕		↑	×									
**Influenza** Huang L., et al., 2024; Zhu R., et al., 2020)	↕						↕	↕	↕						
**RSV[20]** (Huang L., et al., 2024; Okuyan O., et al., 2023; Pan J., et al., 2026)	↕		↕		↕		×	×	×						
**SARS-CoV-2** (Huang L., et al., 2024; Üstündağ Y., et al., 2022; Yilmaz S., et al., 2022)	↕	↕	↕		↑	↑	↓	×	↓	↑	↑	↑	↓	↓	↓
**Mixed bacterial**	↑		↕		↑		↓								

**infection** (Zheng H., et al., 2022; Güneylioğlu M., et al., 2022)															
**Mixed pathogen infection** (Wu J., et al., 2021; Xu M., et al., 2023)	↑						↓								

↑ = Significantly elevated in disease/severe disease; ↓ = Significantly reduced in disease/severe disease; ↕ = Conflicting findings across studies; ╳ = Investigated but not statistically significant; [5]NLR = neutrophil-to-lymphocyte ratio (NLR = neutrophil count/lymphocyte count); [6]MLR = monocyte-to-lymphocyte ratio (MLR = monocyte count/lymphocyte count); [7]PLR = platelet-to-lymphocyte ratio (PLR = platelet count/lymphocyte count); [8]NMLR = neutrophil-monocyte-to-lymphocyte ratio (NMLR = [neutrophil count + monocyte count]/lymphocyte count); [9]SII = systemic immune-inflammation index (SII = neutrophil count x platelet count]/lymphocyte count); [10]SIRI = systemic inflammatory response index (SIRI = [neutrophil count x monocyte count]/lymphocyte count); [11]LMR = lymphocyte-to-monocyte ratio (LMR = lymphocyte count/monocyte count); [12]MPV/PLT = mean platelet volume-to-platelet count (MPV/PLT = mean platelet volume/platelet count); [13]LYM*PLT=lymphocyte multiplied by platelet (LYM*PLT = lymphocyte count x platelet count); [14]dNLR=derived neutrophil-to-lymphocyte ratio (dNLR = neutrophil count/[total leukocytes–neutrophil count]); [15]AISI=aggregate index of systemic inflammation (AISI = [neutrophil count x monocyte count x platelet count]/lymphocyte count); [16]NLPR=neutrophil-to-lymphocyte-platelet ratio (NLPR = neutrophil count/[lymphocyte count x platelet count]); [17]EMR=eosinophil-to-monocyte ratio (EMR = eosinophil count/monocyte count); [18]ELR=eosinophil-to-lymphocyte ratio (ELR = eosinophil count/lymphocyte count); [19]BLR=basophil-to-lymphocyte ratio (BLR = basophil count/lymphocyte count); [20]RSV = respiratory syncytial virus.

Table S2. Etiological differentiation using CBC-derived inflammatory markers across pediatric LRTI pathogens.

Pathogen	NLR[8]		PLR[9]	SII[10]		SIRI[11]	
***Mycoplasma pneumonia* and viral** (Wan Y., et al., 2026; Chen J., et al., 2024; Zhou H., Qian K., 2025)	↑ (MP[4])	↓ (V[6])	↕	↑ (MP[4])	↓ (V[6])	↑ (MP[4])	↓ (V[6])
**Mixed bacterial infection and other pathogen infection** (Gauchan E., Adhikari S, 2016; Omran A., et al., 2022)	↑ (B[5])	↓ (O[7])					

↑ = Significantly elevated in disease/severe disease; ↓ = Significantly reduced in disease/severe disease; ↕ = Conflicting findings across studies; [4]MP = *Mycoplasma pneumonia*; [5]B = bacterial; [6]V = viral; [7]O = other; [8]NLR = neutrophil-to-lymphocyte ratio (NLR = neutrophil count/lymphocyte count); [9]PLR = platelet-to-lymphocyte ratio (PLR = platelet count/lymphocyte count); [10]SII = systemic immune-inflammation index (SII = neutrophil count x platelet count]/lymphocyte count); [11]SIRI = systemic inflammatory response index (SIRI = [neutrophil count x monocyte count]/lymphocyte count).

Table S3. The effectiveness of CBC-derived inflammatory markers in severity assessment of pediatric LRTI pathogens.

Pathogen	NLR[1]	NEUT%[2]	MLR[3]	PLR[4]	NMLR[5]	SII[6]	SIRI[7]	LMR[8]	NPR[9]	LPR[10]	LNR[11]	MPVLR[12]	dNLR[13]	AISI[14]	EMR[15]	ELR[16]
Bordetella pertussis (Li C., et al., 2026)	↑															
Mycobacterium tuberculosis (Ma W., et al., 2024)	↑															
Mycoplasma pneumonia (Li D., et al., 2023; Wang S., et al., 2025; Guo X., et al., 2025)	↑		↑	↑		↑	↑									
Streptococcus pneumoniae (Bao M., et al., 2026)	↑			↑	↓											
**HAdV[17]** (Wang N., et al., 2024; Xu H., et al., 2025)	×	↑		↑	×											
**HMPV[18]** (Liu L., et al., 2026; Guo Y., et al., 2025)	↕			↑	↑											
**RSV[19]** (Okay Z., et al., 2024; Kızılsoy Ö., et al.,2024; Li W., et al., 2026; Lian D., et	↕		×	↕		↑										

al., 2026; Burrack N., et al., 2023)																
**SARS-CoV-2** (Jin Q., et al., 2024; Yazılıtaş F., et al., 2024; Chelcea R., et al., 2024)	↑												↑			
**Mixed bacterial infection** (Han L., et al., 2026)						↑								↑		
**Mixed pathogen infection** (Xu M., et al., 2023; Gambadauro A., et al., 2025; Li C., et al., 2025)	↕		×	×	↕		×	×	×	×	×	×				×
**Mixed viral infection** (Sert S., et al., 2025)	↓		↓	↓			×	×	×				↓		×	

↑ = Significantly elevated in disease/severe disease; ↓ = Significantly reduced in disease/severe disease; ↕ = Conflicting findings across studies; × = Investigated but not statistically significant; [1]NLR = neutrophil-to-lymphocyte ratio (NLR = neutrophil count/lymphocyte count); [2]NEUT% = neutrophil percentage (NEUT% = [neutrophil count/total leukocytes] x 100%); [3]MLR = monocyte-to-lymphocyte ratio (MLR = monocyte count/lymphocyte count); [4]PLR = platelet-to-lymphocyte ratio (PLR = platelet count/lymphocyte count); [5]NMLR = neutrophil-monocyte-to-lymphocyte ratio (NMLR = [neutrophil count + monocyte count]/lymphocyte count); [6]SII = systemic immune-inflammation index (SII = neutrophil count x platelet count]/lymphocyte count); [7]SIRI = systemic inflammatory response index (SIRI = [neutrophil count x monocyte count]/lymphocyte count); [8]LMR = lymphocyte-to-monocyte ratio (LMR = lymphocyte count/monocyte count); [9]NPR = neutrophil-to-platelet ratio (NPR = neutrophil count/platelet count); [10]LPR = lymphocyte-to-platelet ratio (LPR = lymphocyte count/platelet count); [11]LNR = lymphocyte-to-neutrophil ratio (LNR = lymphocyte count/ neutrophil count); [12]MPVLR = mean platelet volume-to-lymphocyte ratio (MPVLR = mean platelet volume/lymphocyte count); [13]dNLR = derived neutrophil-to-lymphocyte ratio (dNLR = neutrophil count/[total leukocytes–neutrophil count]); [14]AISI = aggregate index of systemic inflammation (AISI = [neutrophil count x monocyte count x platelet count]/lymphocyte count); [15]EMR = eosinophil-to-monocyte ratio (EMR = eosinophil count/monocyte count); [16]ELR = eosinophil-to-lymphocyte ratio (ELR = eosinophil count/lymphocyte count); [17]HAdV = human adenovirus; [18]HMPV = human metapneumovirus; [19]RSV = respiratory syncytial virus.

Table S4. The effectiveness of CBC-derived inflammatory markers in predicting the outcome of pediatric LRTI pathogens

Pathogen	NLR[1]	dNLR[2]	LMR[3]	PLR[4]	MLR[5]
**HPIVs[6]** (Chu F., et al., 2023	↑	↑	↓	↑	
**RSV[7]** (Burrack N., et al., 2023)	↑				↑

↑ = Significantly elevated in disease/severe disease; ↓ = Significantly reduced in disease/severe disease; [1]NLR = neutrophil-to-lymphocyte ratio (NLR = neutrophil count/lymphocyte count); [2]dNLR = derived neutrophil-to-lymphocyte ratio (dNLR = neutrophil count/[total leukocytes–neutrophil count]); [3]LMR = lymphocyte-to-monocyte ratio (LMR = lymphocyte count/monocyte count); [4]PLR = platelet-to-lymphocyte ratio (PLR = platelet count/lymphocyte count); [5]MLR = monocyte-to-lymphocyte ratio (MLR = monocyte count/lymphocyte count); [6]HPIVs = human parainfluenza viruses; [7]RSV = respiratory syncytial virus.

Table S5. CBC-derived inflammatory markers in pediatric bacterial LRTI, organized by cell wall structure. Quantitative performance estimates for each marker are provided in Table 3 of the main text.

Etiology	Cell wall characteristics	Study	Main finding
Bordetella pertussis	Gram-negative coccobacillus; outer membrane containing lipooligosaccharide	Li C., et al., 2026	Median NLR[13] (IQR): severe pertussis 0.61 (0.31–1.04) vs non-severe 0.29 (0.19–0.46); p<0.001. NLR[13] was an independent predictor of severe pertussis. Neutrophil count, CRP[14], and PCT[26] were also significantly higher, while lymphocyte percentage was significantly lower in severe pertussis.
		Tang J., et al., 2025	CBC[22]-derived inflammatory markers (NLR[13], MLR[16], SII[17], NMLR[18], SIRI[19]) were significantly elevated and PLR[23] significantly reduced in pertussis. NLR[13] and NMLR[18] showed the best predictive performance for pertussis in children <2 years old, while combined markers further improved diagnostic accuracy (AUC[11] up to 0.913).
		Zhou J., et al., 2025	Pertussis cases had significantly lower NLR[13] [1.23 (0.89–1.87) vs 1.50 (0.96–2.75); p=0.005], lower PLR[23] [81 (62–104) vs 105 (78–136); p<0.001], lower MLR[16] [0.14 (0.11–0.22) vs 0.20 (0.13–0.31); p<0.001], and lower SII[17] [378 (249–605) vs 455 (280–755); p=0.047] compared with PCR[20]-negative children. Meanwhile, no significant

			different between positive and negative result of PCR[20] pertussis group.
Mycobacterium tuberculosis	Acid-fast bacillus, cell wall containing mycolic acid, weakly Gram-positive	Choudhary R., et al., 2019	Children with confirmed TB[1] had significantly elevated MLR[16] at diagnosis (0.407 vs 0.212 in unconfirmed TB[1], p<0.01). MLR[16] independently correlated with active TB[1] disease and declined after treatment, reaching levels similar to non-TB[1] children by week 12. An MLR[16] cut-off of 0.378 demonstrated moderate diagnostic accuracy for confirmed TB[1].
		Kissling M., et al., 2023	Median NLR[13] (IQR): TB[1] disease 2.0 (1.2–2.2), TB[1] exposed 0.8 (0.6–1.3), non-TB[1] LRTI[21] 0.3 (0.1–1.0); p<0.001 (TB[1] vs non-TB[1] LRTI[21]). Median NMLR[18]: TB[1] disease 1.4 (1.2–1.7) vs non-TB[1] LRTI[21] 0.2 (0.1–0.6); p<0.001 (TB vs non-TB LRTI[21]). NLR[13] and NMLR[18] demonstrated diagnostic utility, whereas no significant different of MLR[16] in TB[1] vs non-TB[1] LRTI[21] groups.
		Ma W., et al., 2024	Median NLR[13] was significantly higher in severe TB[1] than non-severe TB[1] (3.24 vs 1.78, p<0.01). Multivariate analysis identified NLR[13], hs-CRP[15] (HR[30] 1.018, p=0.038), age (HR[30] 1.444, p=0.001), symptom duration (HR[30] 0.990, p=0.011), and A/G ratio as independent predictors of severe TB[1]. The nomogram model demonstrated good predictive performance.
		Rees C., et al., 2020	Mean MLR[16]: IGRA[2] negatives vs all converters (0.18, 0.17, p=0.10). Mean NLR[13]: IGRA[2] negatives vs all converters (1.02, 0.88, p=0.08). Mean PLR[23]: IGRA[2] negatives vs all converters (120, 115, p=0.28). None of the biomarkers differ adolescents who developed asymptomatic TB[1] with patients who not infected with TB[1]. These CBC[22]-derived ratios showed poor diagnostic performance and were not useful for predicting or diagnosing incident asymptomatic TB[1] infection.
Mycoplasma pneumoniae	Atypical bacteria (neither Gram-positive nor Gram-negative), no cell wall, pleomorphic, cell membrane containing cholesterol	Chen J., et al., 2024	Compared with influenza infection (207.890), MP[7] infection (145.985) showed significant lower PLR[23] (p<0.001). Eosinophil count demonstrated the highest discriminative ability (AUC[11]=0.824), followed by lymphocyte count and Eosinophil percentage (AUC[11]=0.796).
		Feng M., et al., 2026	Children with PB[3] had significantly higher PLR[23], SII[17], and D-dimer levels than those without PB[3]. The combined PLR[23]–SII[17]–D-dimer model showed the highest

			diagnostic accuracy for predicting PB[3] (AUC[11] = 0.831, sensitivity 84.1%, specificity 72.7%), outperforming individual inflammatory markers.
		Guo X., et al., 2025	Median SII[17] (IQR): SMPP[4] 899.22 (586.98–1360.86) vs non-SMPP[4] 384.67 (242.76–569.28); p<0.001. Median NLR[13]: SMPP[4] 3.13 (1.94–4.03) vs non-SMPP[4] 1.33 (0.83–2.03); p<0.001. SII[17], NLR[13], MLR[16], PLR[23], SIRI[19], CRP[14], and SAA[27] were significantly higher in SMPP[4] than in non-SMPP[4] (all p<0.001). SII[17] was an independent predictor of SMPP[4] and demonstrated superior predictive performance compared with CRP[14].
		Li D., et al., 2023	NLR[13] was an independent predictor of poor outcomes in pediatric MP[7] pneumonia, including necrotizing pneumonia and refractory MPP[8]. Patients with high NLR[13] (≥1.9) had significantly higher risks of NP[5], RMPP[6], ICU[10] admission, prolonged hospitalization, and increased healthcare costs.
		Shao L., et al., 2025	Median NLR[13] (IQR): MP[7] 2.34 (1.60–3.50) vs control 1.07 (0.73–1.34); p<0.001. Median MLR[16]: MP[7] 0.31 (0.22–0.42) vs control 0.15 (0.12–0.19); p<0.001. Median SIRI[19]: MP[7] 1.39 (0.84–2.48) vs control 0.44 (0.30–0.67); p<0.001. MLR[16] and SIRI[19] were independent predictors of MP[7] infection (MLR[16] OR 1.94, 95% CI 1.19–3.18; SIRI[19] OR 1.31, 95% CI 1.18–1.46). SIRI[19] showed the highest diagnostic value.
		Wan Y., et al., 2026	Median NLR[13] (IQR): MPP[8] 1.94 (1.37–2.64), VP[24] 0.95 (0.48–1.53), HC[25] 1.16 (0.86–1.49); p<0.001. NLR[13] cut-off 1.44 differentiated MPP[8] from VP[24] (AUC[11] 0.77, sensitivity 73%, specificity 72%). NLR[13] + YKL-40[28] combined AUC[11] 0.83. NLR[13] independent predictor (OR 1.67, 95% CI 1.08–2.58, p=0.02). PLR[23] did not distinguish MPP[8] from VP[24].
		Wang S., et al., 2024	Children with SMPP[4] had significantly higher SII[17] values than those with non-SMPP[4] (1033.92 vs 422.61, p<0.001). SII[17] independently predicted SMPP[4] and showed superior predictive accuracy compared with NLR[13], PLR[23], and SIRI[19].
		Zhang K., et al., 2026	NLR[13] was significantly elevated in MP[7] infection and was a useful predictor. WBC[29] and NLR[13] were independent predictive markers, while diagnostic agreement

			between RNA and immunogold colloidal testing was poor (Kappa=0.108).
		Zhou H., Qian K., 2025	SIRI[19] in RSV[9] group, median (IQR) = 0.33 (0.21–0.58), was significantly lower than in MP[7] group, median (IQR) = 0.46 (0.26–0.75), with p=0.01. SII[17] in RSV[9] group, median (IQR) = 232.5 (133.1–331.8) also significantly lower than in MP[7] group, median (IQR) = 247.9 (176.3–424.4). Both SIRI[19] and SII[17] showed poor diagnostic value in children 6 months to 1 year but showed satisfying performance in children <6 months and >1 year.
		Zheng H., et al., 2022	NLR[13] was significantly higher and PLR[23] was significantly lower in children with bacterial pneumonia than in those with MP[7] and healthy controls (p<0.05). PLR[23] showed the best diagnostic performance for differentiating bacterial pneumonia from healthy children and from MP[7].
Streptococcus pneumoniae	Gram-positive, lanceolate diplococci, capsule containing polysaccharide	Bao M., et al., 2026	NLR[13] and PLR[23] were significantly higher and PNR was significantly lower in severe SPP[12]. Mean NLR[13] in severe SPP[12] = 2.24 vs non-severe SPP[12] = 1.19, p<0.001. Mean PLR[23] in severe SPP[12] = 103.62 vs non-severe SPP[12] = 87.70, p=0.001. Mean PNR in severe SPP[12] = 54.67 vs non-severe SPP[12] = 70.67, p=0.018.

[1]TB = tuberculosis; [2]IGRA = interferon-gamma release assay; [3]PB = plastic bronchitis; [4]SMPP = severe *Mycoplasma pneumoniae* pneumonia; [5]NP = necrotizing pneumonia; [6]RMPP = refractory *Mycoplasma pneumoniae* pneumonia; [7]MP = *Mycoplasma pneumoniae*; [8]MPP = *Mycoplasma pneumoniae* pneumonia; [9]RSV = respiratory syncytial virus; [10]ICU = intensive care unit; [11]AUC = area under the curve; [12]SPP = *Streptococcus pneumoniae* pneumonia; [13]NLR = neutrophil-to-lymphocyte ratio (NLR = neutrophil count/lymphocyte count); [14]CRP = C-reactive protein; [15]hs-CRP = high sensitivity C-reactive protein; [16]MLR = monocyte-to-lymphocyte ratio (MLR = monocyte count/lymphocyte count); [17]SII = systemic immune-inflammation index (SII = [neutrophil count x platelet count]/lymphocyte count); [18]NMLR = neutrophil-monocyte-to-lymphocyte ratio (NMLR = [neutrophil count + monocyte count]/lymphocyte count); [19]SIRI = systemic inflammatory response index (SIRI = [neutrophil count x monocyte count]/lymphocyte count); [20]PCR = polymerase chain reaction; [21]LRTI = lower respiratory tract infection; [22]CBC = complete blood count; [23]PLR = platelet-to-lymphocyte ratio (PLR = platelet count/lymphocyte count); [24]VP = viral pneumonia; [25]HC = healthy control; [26]PCT = procalcitonin; [27]SAA = serum amyloid A; [28]YKL-40 = chitinase-3-like protein 1; [29]WBC = white blood cell; [30]HR = hazard ratio.

Table S6. CBC-derived inflammatory markers in pediatric viral LRTI, organized by genome type and envelope. Quantitative performance estimates for each marker are provided in Table 4 of the main text.

Etiology	Genetic material	Enveloped	Study	Main finding
Human adenovirus	dsDNA[1], linear	No	Wang N., et al., 2024 [22]	Mean NEUT%[2] = severe vs non-severe adenovirus pneumonia group (57.99, 43.64, p<0.001). In the severe group, the incidences

				of liver injury, coagulation dysfunction, and electrolyte disturbance were significantly higher. Based on statistical analysis, NEUT%[2] with AUC[3] ≥47.60 was risk factor of severe adenovirus pneumonia development.
			Xu H., et al., 2025 [23]	PLR[4] was significantly higher in severe adenovirus pneumonia group compared with non-severe ($p= 0.0184$). Whereas, NLR[5] and PNR[6] did not show significant different between those groups.
Human metapneumovirus	(-)ssRNA[7], non-segmented	Yes	Guo Y., et al., 2025 [24]	Mean NLR[5] = severe vs non-severe HMPV[8] infection (2.2, 1.1, $p<0.01$). Mean PNR[6] = severe vs non-severe HMPV[8] infection (106.8, 133, $p=0.022$). Mean PLR[4] = severe vs non-severe HMPV[8] infection (129.8, 93.8, $p<0.01$). All markers were reliable as HMPV[8] severity predictor.
			Liu L., et al., 2026 [25]	Mean NLR[5] in HMPV[8] infection = severe vs mild CAP[9] (315, 163, $p<0.01$).
Human parainfluenza viruses	(-)ssRNA[7], non-segmented	Yes	Chu F., et al., 2023 [26]	Elevated NLR[5], dNLR[10], PLR[4], and reduced LMR[11] significantly associated with PICU[12] admission. High PLR[4] level ($p=0.018$) independently being risk factors for PICU[12] admission.
RSV[13]	(-)ssRNA[7], non-segmented	Yes	Okay Z., et al., 2024 [27]	Mean NLR[5] significantly different among groups ($p<0.001$): mild (0.52), moderate (0.54), severe (0.87), healthy control (1.14). NLR[5] was higher in severe RSV[13], although not as high as control group.
			Okuyan O., et al., 2023 [28]	Median NLR[5]: RSV[13] negative vs RSV[13] positive (1.37, 0.81, $p<0.001$). Median PLR[4]: RSV[13] negative vs RSV[13] positive (119.31, 72.17, $p<0.001$). Median SII[14]: RSV[13] negative vs RSV[13] positive (449.5, 243.4, $p<0.001$). Decreasing values of NLR[5], PLR[4], and SII[14] indicated RSV[13] infection.
			Pan J., et al., 2026 [29]	Elevated NLR[5] had a positive influence on the severity

				prediction, median (IQR[29]) NLR[5] in severe cases = 0.64 (0.33, 1.44); p<0.001.
			Burrack N., et al., 2023 [30]	This study categorized subjects into four quartiles based on NLR[5] and MLR[15] values (low=Q1 until high=Q4). The incidence of PICU[12] admission was higher in MLR[15]-Q4 (9.4%) and the PICU[12] length of stay was longer (median=4 days), similar to NLR[5]-Q4 (PICU[12] admission = 5.3%; LOS[30] median = 4 days). Increased both MLR[15] and NLR[5] were severity risk factors, showed by the values of RR[16] and HR[17], also co-infections incidence.
			Li W., et al., 2026 [31]	Mean NLR[5] = severe vs non-severe RSV[13] infection (0.86, 0.66, p=0.001)
			Lian D., et al., 2026 [32]	Median NLR[5] = with and without bacterial co-infection (1.64, 0.54, p<0.001). Mean PLR[4] = with and without bacterial co-infection (103, 74, p<0.001). A multivariable logistic regression analysis showed beta value of NLR[5] = 0.758 as independent bacterial co-infection predictor in training cohort.
			Huang L., et al., 2024 [39]	Mean NLR[5] in children below 6 months: SARS-CoV-2[27], influenza A, RSV[13], and healthy control (1.59, 1.16, 1.93, 1.42, p=0.004). Mean NLR[5] in children 6–24 months: SARS-CoV-2[27], influenza A, RSV[13], and healthy control (0.52, 0.32, 0.41, 0.35, p=0.001). Mean NLR[5] in children 2–6 years: SARS-CoV-2[27], influenza A, RSV[13], and healthy control (1.04, 0.83, 0.91, 0.63, p=0.001). Mean NLR[5] in children 6–18 years: SARS-CoV-2[27], influenza A, RSV[13], and healthy control (1.65, 0.90, 1.25, 1.09, p=0.011).
			Kızılsoy Ö., et al., 2024 [33]	Median NLR[5] = mild vs severe RSV[13] bronchiolitis (0.69, 3.24, p=0.030). Median PLR[4] = mild vs severe RSV[13] bronchiolitis (84.66, 166.53, p=0.204). Median

				SII[14] = mild vs severe RSV[13] bronchiolitis (295.03, 1175.64, p=0.30).
SARS-CoV-2[27]	(+)ssRNA[7], non-segmented	Yes	Yilmaz S., et al., 2022 [34]	Mean NLR[5]: COVID-19[28]-positive vs COVID-19[28]-negative (1.93, 2.44, p=0.133). Mean MLR[15]: COVID-19[28]-positive vs COVID-19[28]-negative (0.316, 0.326, p=0.805). Mean ELR[18]: COVID-19[28]-positive vs COVID-19[28]-negative (0.039, 0.075, p=0.005). Mean BLR[19]: COVID-19[28]-positive vs COVID-19[28]-negative (0.008, 0.013, p<0.001). Mean PLR[4]: COVID-19[28]-positive vs COVID-19[28]-negative (115.8, 116.6, p=0.931). Mean MPV/PLT[20]: COVID-19[28]-positive vs COVID-19[28]-negative (3.63, 3.16, p=0.159).
			Üstündağ Y., et al., 2022 [35]	Median NLR[5]: COVID-19[28] patients vs healthy control (1.7, 1.3, p=0.03). Median PLR[4]: COVID-19[28] patients vs healthy control (123, 109, p=0.46). Median MLR[15]: COVID-19[28] patients vs healthy control (0.27, 0.16, p<0.001). Median dNLR[10]: COVID-19[28] patients vs healthy control (1.3, 1.0, p=0.05). Median AISI[21]: COVID-19[28] patients vs healthy control (264, 155, p<0.001). Median NLPR[22]: COVID-19[28] patients vs healthy control (0,0066, 0.0046, p<0.001). Median SII[14]: COVID-19[28] patients vs healthy control (404, 365, p=0.034). Median SIRI[23]: COVID-19[28] patients vs healthy control (0.99, 0.54, p<0.001). Median EMR[24]: COVID-19[28] patients vs healthy control (0.11, 0.34, p<0.001)
			Yazılıtaş F., et al., 2024 [36]	Mean NLR[5] in pediatric COVID-19[28] patients: AKI[25] vs non-AKI[25] (1.4, 4.1, p=0.001)
			Chelcea R., et al., 2024 [37]	Mean NLR[5] in pediatric COVID-19[28] patients: pneumonia vs non-pneumonia (8.80, 5.36, p=0.008). Mean dNLR[10] in pediatric COVID-19[28] patients: pneumonia vs non-pneumonia (1.49, 0.73, p=0.006).

			Huang L., et al., 2024 [39]	Mean NLR[5] in children below 6 months: SARS-CoV-2[27], influenza A, RSV[13], and healthy control (1.59, 1.16, 1.93, 1.42, p=0.004). Mean NLR[5] in children 6–24 months: SARS-CoV-2[27], influenza A, RSV[13], and healthy control (0.52, 0.32, 0.41, 0.35, p=0.001). Mean NLR[5] in children 2–6 years: SARS-CoV-2[27], influenza A, RSV[13], and healthy control (1.04, 0.83, 0.91, 0.63, p=0.001). Mean NLR[5] in children 6–18 years: SARS-CoV-2[27], influenza A, RSV[13], and healthy control (1.65, 0.90, 1.25, 1.09, p=0.011).
			Jin Q., et al., 2024 [38]	Mean MLR[15]: severe vs non-severe COVID-19[28] (0.6197, 0.5450, p=0.193) Mean PLR[4]: severe vs non-severe COVID-19[28] (174.2302, 158.3351, p=0.352). Mean NLR[5]: severe vs non-severe COVID-19[28] (4.9484, 3.0939, p=0.003).
Influenza A	(-)ssRNA[7], segmented	Yes	Huang L., et al., 2024 [39]	Mean NLR[5] in children below 6 months: SARS-CoV-2[27], influenza A, RSV[13], and healthy control (1.59, 1.16, 1.93, 1.42, p=0.004). Mean NLR[5] in children 6–24 months: SARS-CoV-2[27], influenza A, RSV[13], and healthy control (0.52, 0.32, 0.41, 0.35, p=0.001). Mean NLR[5] in children 2–6 years: SARS-CoV-2[27], influenza A, RSV[13], and healthy control (1.04, 0.83, 0.91, 0.63, p=0.001). Mean NLR[5] in children 6–18 years: SARS-CoV-2[27], influenza A, RSV[13], and healthy control (1.65, 0.90, 1.25, 1.09, p=0.011).
			Zhu R., et al., 2020 [40]	NLR[5] was significantly higher in the A+ group than in H controls for younger children (p<0.01), reflecting age-specific immune dynamics. Within the 1–6 y cohort, the A+ group also demonstrated significantly lower LMR[11] and LYM*PLT[26], alongside an elevated MPV/PLT[20] ratio compared with

				both A-/B- and H groups (p<0.01).<br><br>In patients aged>6 y, NLR[5] was significantly higher, and LMR[11] significantly lower in the A+ group compared with all other cohorts (p<0.01). Similar to the younger cohort, LYM*PLT[26] remained significantly lower, while the MPV/PLT[20] ratio was significantly higher specifically against the H group (p<0.01), showing distinct hematological shifts across different age brackets.
Influenza B	(-)ssRNA[7], segmented	Yes	Zhu R., et al., 2020 [40]	Similarly, NLR[5] was significantly higher in the B+ group than in H controls for the 1–6 y cohort (p<0.01). This younger group also exhibited significantly lower LMR[11] alongside an elevated MPV/PLT[20] ratio compared with H controls (p<0.01), while LYM*PLT[26] was significantly decreased against both A-/B- (p<0.05) and H groups (p<0.01).<br><br>In patients age >6 y, the B+ group maintained a significantly higher NLR[5] and lower LMR[11] compared with H controls and other cohorts (p<0.01). Furthermore, both MPV/PLT[20] and LYM*PLT[26] mirrored the trends of the younger cohort, showing significantly higher and lower values respectively when compared with the H group (p<0.01), indicating consistent biomarker patterns across age groups.

[1]dsDNA = double-stranded deoxyribonucleic acid; [2]NEUT% = neutrophil percentage (NEUT% = [neutrophil count/total leukocytes] x 100%); [3]AUC = area under the curve; [4]PLR = platelet-to-lymphocyte ratio; [5]NLR = neutrophil-to-lymphocyte ratio (NLR = neutrophil count/lymphocyte count); [6]PNR = platelet-to-neutrophil ratio (PNR = platelet count/neutrophil count); [7]ssRNA = single-stranded ribonucleic acid; [8]HMPV = human metapneumovirus; [9]CAP = community-acquired pneumonia; [10]dNLR = derived neutrophil-to-lymphocyte ratio (dNLR = neutrophil count/[total leukocytes–neutrophil count]); [11]LMR = lymphocyte-to-monocyte ratio (LMR = lymphocyte count/monocyte count); [12]PICU = pediatric intensive care unit; [13]RSV = respiratory syncytial virus; [14]SII = systemic immune-inflammation index (SII = [neutrophil count x platelet count]/lymphocyte

count); [15]MLR = monocyte-to-lymphocyte ratio; [16]RR = respiratory rate; [17]HR = heart rate; [18]ELR = eosinophil-to-lymphocyte ratio; [19]BLR = basophil-to-lymphocyte ratio; [20]MPV/PLT = mean platelet volume-to-platelet count (MPV/PLT = mean platelet volume/platelet count); [21]AISI = aggregate index of systemic inflammation; [22]NLPR = neutrophil-to-lymphocyte-platelet ratio; [23]SIRI = systemic inflammatory response index (SIRI = [neutrophil count x monocyte count]/lymphocyte count); [24]EMR = eosinophil-to-monocyte ratio; [25]AKI = acute kidney injury; [26]LYM*PLT = lymphocyte multiplied by platelet (LYM*PLT = lymphocyte count x platelet count); [27]SARS-CoV-2 = severe acute respiratory syndrome coronavirus 2; [28]COVID-19 = coronavirus disease 2019; [29]IQR = interquartile range; [30]LOS = length of stay

Table S7. CBC-derived inflammatory markers in mixed-pathogen pediatric LRTI. Quantitative performance estimates for each marker are provided in Table 5 of the main text.

**Etiologies**	**Study**	**Main finding**
Mixed pathogen infection, dominated by: *Streptococcus pneumoniae, Streptococcus pyogenes*, and *Staphylococcus aureus*.	Güneylioğlu M., et al., 2022	Mean SII[3]: empyema vs parapneumonic effusion (6899.98, 1902, p=0.009). Mean NLR[7]: empyema vs parapneumonic effusion (17.45, 5.87, p=0.011). Mean LMR[4]: empyema vs parapneumonic effusion: (1.65, 3.77, p<0.001). Mean PLR[5]: empyema vs parapneumonic effusion (361.35, 171.58, p=0.004).
Mixed bacterial infection: *Haemophilus influenzae, Streptococcus pneumoniae, Staphylococcus aureus*, and *Moraxella catarrhalis*	Han L., et al., 2026	Children who developed hospital-acquired OM[11] had significantly higher inflammatory markers, including SII[3] and AISI[6], and lower RBC[12] and hemoglobin levels. Both indices were independent risk factors for OM[11]. The AISI[6]-based model demonstrated slightly better predictive performance than the SII[3]-based model, indicating that elevated systemic inflammation is associated with increased risk of OM[11] in pediatric bacterial pneumonia.
Mycoplasma pneumoniae, and bacterial pneumonia	Zheng H., et al., 2022	NLR[7] was significantly higher and PLR[5] was significantly lower in children with bacterial pneumonia than in those with MP[1] and healthy controls (p<0.05). PLR[5] showed the best diagnostic performance for differentiating bacterial pneumonia from healthy children (AUC[10] 0.898, sensitivity 88.4%, specificity 84.1%) and from MP[1] (AUC[10] 0.803, sensitivity 70.5%, specificity 84.6%).
Bacterial pneumonia, and other pathogens related with pneumonia	Gauchan E., Adhikari S, 2016	Mean NLR[7]: bacterial pneumonia vs non-bacterial pneumonia (1.8, 1.67, p=0.468)
Bacterial and viral pneumonia	Omran A., et al., 2022	LUS and NLR[7] effectively differentiated bacterial from viral pneumonia in children <2 years old. Bacterial pneumonia was characterized by larger subpleural consolidations (>1 cm) and air bronchograms, whereas viral pneumonia was associated with multiple B-lines and smaller consolidations (<1 cm). LUS demonstrated higher diagnostic accuracy than chest radiography for both bacterial (sensitivity 88.2%, specificity 100%)

		and viral pneumonia (sensitivity 83.3%, specificity 85.7%), and diagnostic performance improved further when combined with NLR[7]. Mean NLR[7]: bacterial vs viral pneumonia (1.8, 0.66, $p<0.0001$)
Viral bronchiolitis, predominantly RSV, Rhinovirus, Metapneumovirus, Adenovirus, SARS-CoV-2, Influenza, Parainfluenza, Enterovirus, and Coronavirus OC43	Gambadauro A., et al., 2025	Mean PLR[5]: prolonged LOS 98.9 vs non-prolonged LOS 134.4; p=0.003. Mean MLR: prolonged LOS 0.14 vs non-prolonged LOS 0.19; p=0.01. Mean PNR[8]: prolonged LOS 99.7 vs non-prolonged LOS 140.5; p=0.03. NLR[7] showed no significant association with LOS (1.23 vs 1.31; p=0.72) or other severity outcomes
Mixed pathogen infection	Li C., et al., 2025	Mean PNR[8] among neonates: pneumonia vs pneumonia with sepsis (91.18, 36.69). The proportion of neonate sepsis was higher in the low PNR[8] group compared with the high PNR[8] group ($p<0.001$).
Mixed pathogen infection	Wu J., et al., 2021	LMR[4] was significantly higher and NLR[7] significantly lower in pneumonia than URTI. LMR[4] showed the highest diagnostic utility, followed by NLR[7]. For viral pneumonia, NLR[7] achieved AUC[10] 0.86 and LMR[4] AUC[10] 0.80; for bacterial pneumonia, LMR[4] achieved AUC[10] 0.77 and NLR[7] AUC[10] 0.71. Combining LMR[4] and NLR[7] with age and clinical symptoms improved discrimination (AUC[10] 0.84 overall, 0.95 viral, 0.86 bacterial).
Mixed pathogen infection	Xu M., et al., 2023	NLR[7] cut-off 4.15 for CAP[2] in asthmatic children: AUC[10] 0.711 (95% CI 0.670–0.751), sensitivity 56.9%, specificity 90.1%, PPV[14] 85.1%, NPV[15] 67.7%. NLR[7]>4.15 also predicted asthmatic exacerbation (HR[13] 2.91, 95% CI 1.83–3.96) and unimproved CAP[2] (HR[13] 3.38, 1.66–5.10).
Viral pneumonia, predominantly RSV, influenza A, influenza B, adenovirus, parainfluenza, human rhinovirus, enterovirus, human metapneumovirus, and human bocavirus	Sert S., et al., 2025	Pediatric respiratory severity score (PRESS) was inversely correlated with NLR[7] ($r = –0.337$, $p<0.0001$); NMR[9] ($r = –0.260$, $p< 0.0001$), PLR[5] ($r = –0.215$, $p= 0.003$), and MPVLR ($r = –0.441$, $p< 0.0001$). The rest of the blood markers were not significantly correlated with PRESS.

[1]MP = *Mycoplasma pneumoniae*; [2]CAP = community-acquired pneumonia; [3]SII = systemic immune-inflammation index (SII = [neutrophil count x platelet count]/lymphocyte count); [4]LMR = lymphocyte-to-monocyte ratio (LMR = lymphocyte count/monocyte count); [5]PLR = platelet-to-lymphocyte ratio (PLR = platelet count/lymphocyte count); [6]AISI = aggregate index of systemic inflammation (AISI = [neutrophil count x monocyte count x platelet count]/lymphocyte count); [7]NLR = neutrophil-to-lymphocyte ratio (NLR = neutrophil count/lymphocyte count); [8]PNR = platelet-to-neutrophil ratio (PNR = platelet count/neutrophil count); [9]NMR = neutrophil-to-monocyte ratio (NMR = neutrophil count/monocyte count); [10]AUC = area under curve; [11]OM = otitis media; [12]RBC = red blood cell; [13]HR = hazard ratio; [14]PPV = positive predictive values; [15]NPV = negative predictive values.

Table S8. The Boolean codes for PubMed advanced search. The final PubMed search was conducted on 9 August 2026.

**Population**	("Child"[Mesh] OR "Infant"[Mesh] OR "Adolescent"[Mesh] OR “child”[tiab] OR “pediatric”[tiab] OR “pediatric”[tiab] OR “infant”[tiab] OR “toddler”[tiab] OR “adolescent”[tiab] OR “newborn”[tiab])
**Exposure #1**	("Pneumonia"[Mesh] OR "Bronchiolitis"[Mesh] OR "Bronchitis"[Mesh] OR "Respiratory Tract Infections"[Mesh] OR "lower respiratory tract infection”[tiab] OR “LRTI”[tiab] OR “pneumonia”[tiab] OR “bronchopneumonia”[tiab] OR “bronchiolitis”[tiab] OR “bronchitis”[tiab] OR "community-acquired pneumonia”[tiab] OR "acute lower respiratory infection”[tiab] OR “ALRI”[tiab])
**Exposure #2**	The Boolean codes depending on the etiology being investigated (e.g. *Mycobacterium tuberculosis*)<br><br>("Tuberculosis, Pulmonary"[Mesh] OR "Tuberculosis"[Mesh] OR "Mycobacterium tuberculosis"[Mesh] OR "pulmonary tuberculosis"[tiab] OR “tuberculosis"[tiab] OR “TB"[tiab] OR “PTB"[tiab] OR "*Mycobacterium tuberculosis*"[tiab] OR “MTB"[tiab])<br>The corresponding pathogen-specific Boolean codes for the other etiologies were:<br>*Bordetella pertussis*: ("Whooping Cough"[Mesh] OR "Bordetella pertussis"[Mesh] OR "pertussis"[tiab] OR "whooping cough"[tiab] OR "*Bordetella pertussis*"[tiab] OR "B. pertussis"[tiab])<br>*Mycoplasma pneumoniae*: ("Pneumonia, Mycoplasma"[Mesh] OR "Mycoplasma pneumoniae"[Mesh] OR "Mycoplasma pneumoniae"[tiab] OR "*Mycoplasma pneumoniae* pneumonia"[tiab] OR "MPP"[tiab] OR "atypical pneumonia"[tiab])<br>*Streptococcus pneumoniae*: ("Pneumonia, Pneumococcal"[Mesh] OR "Streptococcus pneumoniae"[Mesh] OR "Streptococcus pneumoniae"[tiab] OR "pneumococcal pneumonia"[tiab] OR "pneumococcus"[tiab])<br>Human adenovirus: ("Adenovirus Infections, Human"[Mesh] OR "Adenoviruses, Human"[Mesh] OR "adenovirus"[tiab] OR "adenoviral"[tiab] OR "HAdV"[tiab])<br>Human metapneumovirus: ("Metapneumovirus"[Mesh] OR "human metapneumovirus"[tiab] OR "metapneumovirus"[tiab] OR "HMPV"[tiab] OR "hMPV"[tiab])<br>Human parainfluenza viruses: ("Paramyxoviridae Infections"[Mesh] OR "Parainfluenza Virus 1, Human"[Mesh] OR "Parainfluenza Virus 2, Human"[Mesh] OR "Parainfluenza Virus 3, Human"[Mesh] OR "parainfluenza"[tiab] OR "HPIV"[tiab] OR "HPIVs"[tiab])<br>Influenza: ("Influenza, Human"[Mesh] OR "Influenza A virus"[Mesh] OR "Influenza B virus"[Mesh] OR "influenza"[tiab] OR "influenza A"[tiab] OR "influenza B"[tiab])<br>Respiratory syncytial virus: ("Respiratory Syncytial Virus Infections"[Mesh] OR "Respiratory Syncytial Virus, Human"[Mesh] OR "respiratory syncytial virus"[tiab] OR "RSV"[tiab] OR "bronchiolitis"[tiab])<br>SARS-CoV-2: ("COVID-19"[Mesh] OR "SARS-CoV-2"[Mesh] OR "COVID-19"[tiab] OR "SARS-CoV-2"[tiab] OR "coronavirus disease 2019"[tiab] OR "2019-nCoV"[tiab])
**Outcome**	("Blood Cell Count"[Mesh] OR "Leukocyte Count"[Mesh] OR "Neutrophils"[Mesh] OR "Lymphocytes"[Mesh] OR "Monocytes"[Mesh] OR "Blood Platelets"[Mesh] OR "Inflammation Mediators"[Mesh] OR "complete blood count"[tiab] OR “CBC” [tiab] OR “leukocyte” [tiab] OR “WBC”[tiab] OR “neutrophil”[tiab] OR “lymphocyte”[tiab] OR “monocyte”[tiab] OR “platelet”[tiab] OR “AISI”[tiab] OR "aggregate index of systemic inflammation”[tiab] OR “BLR”[tiab] OR "basophil-to-lymphocyte ratio”[tiab] OR “dNLR”[tiab] OR "derived neutrophil-to-lymphocyte ratio”[tiab] OR “ELR”[tiab] OR "eosinophil-to-lymphocyte ratio”[tiab] OR “LMR”[tiab] OR "lymphocyte-to-monocyte ratio”[tiab] OR “MLR”[tiab] OR "monocyte-to-lymphocyte ratio”[tiab] OR “MPR”[tiab] OR "mean platelet volume-to-platelet ratio”[tiab] OR “NLR”[tiab] OR "neutrophil-to-lymphocyte ratio”[tiab] OR “NMR”[tiab] OR "neutrophil-to-monocyte ratio”[tiab] OR “PIV”[tiab] OR "pan-immune-inflammation value”[tiab] OR “PLR”[tiab] OR "platelet-to-lymphocyte ratio”[tiab] OR “PMR”[tiab] OR "platelet-to-monocyte ratio”[tiab] OR “PWR”[tiab] OR "platelet-to-white blood cell ratio”[tiab] OR “RLR”[tiab] OR “red cell distribution width-to-lymphocyte ratio”[tiab] OR “RPR”[tiab] OR "red cell distribution width-to-platelet ratio”[tiab] OR “SII”[tiab] OR

	"systemic immune-inflammation index"[tiab] OR "SIRI"[tiab] OR "systemic inflammation response index"[tiab] OR "systemic inflammatory response index"[tiab] OR "hematological inflammatory biomarker"[tiab] OR "hematologic inflammatory biomarker"[tiab] OR "CBC-derived inflammatory marker"[tiab] OR "blood cell-derived inflammatory marker"[tiab])
**Entry term**	("Child"[Mesh] OR "Infant"[Mesh] OR "Adolescent"[Mesh] OR "child"[tiab] OR "pediatric"[tiab] OR "pediatric"[tiab] OR "infant"[tiab] OR "toddler"[tiab] OR "adolescent"[tiab] OR "newborn"[tiab])<br>AND<br>("Pneumonia"[Mesh] OR "Bronchiolitis"[Mesh] OR "Bronchitis"[Mesh] OR "Respiratory Tract Infections"[Mesh] OR "lower respiratory tract infection"[tiab] OR "LRTI"[tiab] OR "pneumonia"[tiab] OR "bronchopneumonia"[tiab] OR "bronchiolitis"[tiab] OR "bronchitis"[tiab] OR "community-acquired pneumonia"[tiab] OR "acute lower respiratory infection"[tiab] OR "ALRI"[tiab])<br>AND<br>("Tuberculosis, Pulmonary"[Mesh] OR "Tuberculosis"[Mesh] OR "Mycobacterium tuberculosis"[Mesh] OR "pulmonary tuberculosis"[tiab] OR "tuberculosis"[tiab] OR "TB"[tiab] OR "PTB"[tiab] OR "*Mycobacterium tuberculosis*"[tiab] OR "MTB"[tiab])<br>AND<br>("Blood Cell Count"[Mesh] OR "Leukocyte Count"[Mesh] OR "Neutrophils"[Mesh] OR "Lymphocytes"[Mesh] OR "Monocytes"[Mesh] OR "Blood Platelets"[Mesh] OR "Inflammation Mediators"[Mesh] OR "complete blood count"[tiab] OR "CBC" [tiab] OR "leukocyte" [tiab] OR "WBC"[tiab] OR "neutrophil"[tiab] OR "lymphocyte"[tiab] OR "monocyte"[tiab] OR "platelet"[tiab] OR "AISI"[tiab] OR "aggregate index of systemic inflammation"[tiab] OR "BLR"[tiab] OR "basophil-to-lymphocyte ratio"[tiab] OR "dNLR"[tiab] OR "derived neutrophil-to-lymphocyte ratio"[tiab] OR "ELR"[tiab] OR "eosinophil-to-lymphocyte ratio"[tiab] OR "LMR"[tiab] OR "lymphocyte-to-monocyte ratio"[tiab] OR "MLR"[tiab] OR "monocyte-to-lymphocyte ratio"[tiab] OR "MPR"[tiab] OR "mean platelet volume-to-platelet ratio"[tiab] OR "NLR"[tiab] OR "neutrophil-to-lymphocyte ratio"[tiab] OR "NMR"[tiab] OR "neutrophil-to-monocyte ratio"[tiab] OR "PIV"[tiab] OR "pan-immune-inflammation value"[tiab] OR "PLR"[tiab] OR "platelet-to-lymphocyte ratio"[tiab] OR "PMR"[tiab] OR "platelet-to-monocyte ratio"[tiab] OR "PWR"[tiab] OR "platelet-to-white blood cell ratio"[tiab] OR "RLR"[tiab] OR "red cell distribution width-to-lymphocyte ratio"[tiab] OR "RPR"[tiab] OR "red cell distribution width-to-platelet ratio"[tiab] OR "SII"[tiab] OR "systemic immune-inflammation index"[tiab] OR "SIRI"[tiab] OR "systemic inflammation response index"[tiab] OR "systemic inflammatory response index"[tiab] OR "hematological inflammatory biomarker"[tiab] OR "hematologic inflammatory biomarker"[tiab] OR "CBC-derived inflammatory marker"[tiab] OR "blood cell-derived inflammatory marker"[tiab])
